# Performance of metabonomic serum analysis for diagnostics in paediatric tuberculosis

**DOI:** 10.1038/s41598-020-64413-6

**Published:** 2020-04-29

**Authors:** Nicholas J. Andreas, Robindra Basu Roy, Maria Gomez-Romero, Verena Horneffer-van der Sluis, Matthew R. Lewis, Stephane S. M. Camuzeaux, Beatriz Jiménez, Joram M. Posma, Leopold Tientcheu, Uzochukwu Egere, Abdou Sillah, Toyin Togun, Elaine Holmes, Beate Kampmann

**Affiliations:** 1 Centre for International Child Health, Department of Paediatrics, Imperial College London, St. Mary’s Hospital, Praed Street, London, W2 1NY United Kingdom; 2 Division of Computational and Systems Medicine, Department of Surgery and Cancer, Imperial College London, Sir Alexander Fleming Building, South Kensington, London, SW7 2AZ United Kingdom; 3 MRC-NIHR National Phenome Centre, Department of Surgery and Cancer, Imperial College London, IRDB Building, Du Cane Road, London, W12 0NN United Kingdom; 4 Clinical Phenotyping Centre, Department of Surgery and Cancer, Imperial College London, Sir Alexander Fleming Building, South Kensington, London, SW7 2AZ United Kingdom; 5 MRC Unit The Gambia at the London School of Hygiene and Tropical Medicine, Vaccines and Immunity Theme, Atlantic Road, Fajara, The Gambia; 6 The Vaccine Centre, Department of Clinical Research, Faculty of Infectious and Tropical Diseases, London School of Hygiene and Tropical Medicine, Keppel Street, London, WC1E 7HT United Kingdom

**Keywords:** Lipidomics, Metabolomics, Diagnostic markers, Paediatric research, Molecular medicine

## Abstract

We applied a metabonomic strategy to identify host biomarkers in serum to diagnose paediatric tuberculosis (TB) disease. 112 symptomatic children with presumptive TB were recruited in The Gambia and classified as bacteriologically-confirmed TB, clinically diagnosed TB, or other diseases. Sera were analysed using ^1^H nuclear magnetic resonance (NMR) spectroscopy and mass spectrometry (MS). Multivariate data analysis was used to distinguish patients with TB from other diseases. Diagnostic accuracy was evaluated using Receiver Operating Characteristic (ROC) curves. Model performance was tested in a validation cohort of 36 children from the UK. Data acquired using ^1^H NMR demonstrated a sensitivity, specificity and Area Under the Curve (AUC) of 69% (95% confidence interval [CI], 56–73%), 83% (95% CI, 73–93%), and 0.78 respectively, and correctly classified 20% of the validation cohort from the UK. The most discriminatory MS data showed a sensitivity of 67% (95% CI, 60–71%), specificity of 86% (95% CI, 75–93%) and an AUC of 0.78, correctly classifying 83% of the validation cohort. Amongst children with presumptive TB, metabolic profiling of sera distinguished bacteriologically-confirmed and clinical TB from other diseases. This novel approach yielded a diagnostic performance for paediatric TB comparable to that of Xpert MTB/RIF and interferon gamma release assays.

## Introduction

Diagnosis of tuberculosis (TB) in children remains challenging, and developing better diagnostics is a priority^1–3^. Diagnostic tools based on detecting *Mycobacterium tuberculosis (M.tb)*, including smear microscopy, culture, and Xpert MTB/RIF perform well in adults. However, they fail to diagnose two-thirds of children with suspected TB, due to the paucibacillary nature of paediatric disease^2–6^. Age, as well as the development of the immune system, further complicates assessment, as the performance of tests for latent TB infection varies with age^2,7,8^. The non-specific clinical presentation of TB in children presents a further diagnostic challenge^3,9^. In low-and middle income countries, where the majority of the burden of TB disease lies, approximately 40% of patients are incorrectly diagnosed^10^. Consequently, many children are not appropriately treated, and over 210,000 children are estimated to die every year^11^.

Developing novel diagnostics is a key component of the global End TB Strategy, and the goal of zero childhood TB deaths^1,12^. Host-based biomarkers of TB show promise, with gene expression signatures and flow cytometry techniques potentially capable of distinguishing TB cases from controls, including in children, but published studies are often small and prone to bias^13–17^. While these data provide confidence in the concept of a host-derived diagnostic signature, both gene expression profiling and flow cytometric measurements currently remain far removed from a point-of-care test. Protein-based diagnostics could be easier to translate into a point-of-care test, although paediatric data is sparse^18^. A recent systematic review of TB biomarker data published since 2010 in over 400 scientific papers shows the overall activity in the field but contained only 6% of data relating to studies in children^19^.

Here, we report the application of ^1^H Nuclear Magnetic Resonance (NMR) spectroscopy and untargeted Ultra-performance Liquid Chromatography-Mass Spectrometry (UPLC-MS) based assays, to identify diagnostic biomarkers of TB disease amongst children with presumptive TB in The Gambia. We validated our findings in a distinct cohort of children with TB from the UK. Novel host biomarkers of paediatric TB were detected and specific metabolites identified, showing promising diagnostic potential on a readily available biofluid.

## Results

### Discovery cohort patient characteristics

The demographic characteristics, case classification, and number of samples analysed on each analytical platform are shown in Table 1. 22 children had bacteriologically confirmed TB and 33 fulfilled the category of clinically diagnosed TB. There were no significant differences in weight and age between the different case classifications. All children were HIV negative.

**Table 1:** The mean weight, age, and sex of participants as well as number for each patient diagnosis is given, range in brackets. Data refers to samples analysed using lipidomics.

Characteristic	Discovery Cohort						Validation cohort			
	Other Diseases (n = 57)		Bacteriologically confirmed TB (n = 22)		Clinically diagnosed TB (n = 33)		Bacteriologically confirmed TB (n = 14)		Clinically diagnosed TB (n = 22)	
Age (years)	6.29 (0.7–14)		5.4 (0.4–13)		5.3 (0.3–12)		8 (1–15)		6.0 (1–13)	
Sex	M	F	M	F	M	F	M	F	M	F
	29	28	7	15	15	18	7	7	11	11
Mean Weight^53^	18.8 (7–42)		18.7 (5.1–52.2)		16.9 (5.1–34.7)		32.4 (5.0–64.1)		23.5 (9.2–57.8)	
Positive tuberculin skin test — no. (%)*	10 (18%)		14/21 (66%)		11 (33%)		11 (79%)		17 (77%)	
Positive IGRA — no/total no. (%)	28 (49%)		13/15 (87%)		23/32 (72%)		11/12 (92%)**		16/21 (76%)**	
Culture-positive TB — no. (%)	0		19 (86%)		0		14 (100%)		0	
Sputum positive TB — no. (%)	0		4 (18%)		0		3/13 (23%)		0	
Xpert MTB/RIF positive TB — no. (%)	0		10 (45%)		0		—		—	
Direct referrals to paediatric TB clinic in The Gambia	18 (32%)		8 (36%)		8/32 (25%)		—		—	
Identified as symptomatic through contact tracing in The Gambia	39 (68%)		14 (64%)		24/32 (75%)		—		—	
Samples analysed by lipidomics ESI+ (total 112)	57/57		22/22		33/33		14/14		22/22	
Samples analysed by lipidomics ESI− (total 112)	57/57		22/22		33/33		14/14		22/22	
Samples analysed by NMR (total 95)	49/57		17/22		29/33		9/14		19/22	
Samples analysed by HILIC (total 108)	56/57		21/22		31/33		13/14		22/22	

*A positive result on the tuberculin skin test was defined according to guidelines from the World Health Organization as an induration of 10 mm or more.

**One result was indeterminate in the clinically diagnosed group and two in the bacteriologically-confirmed group.

### Combining clinically diagnosed and bacteriologically-confirmed TB participants

The metabolic profiles of the 33 participants with clinically diagnosed TB were compared to the 22 participants with bacteriologically-confirmed TB. Unsupervised (blinded) Principal Component Analyses (PCA) was used to identify the source of the greatest variation in the data, and to establish whether there were obvious groupings in the scores plot of participants (similar profiles are closer together).

Supervised (unblinded) orthogonal partial least squares-discriminant analysis (OPLS-DA) was then used to establish whether there were systematic differences in the metabolic profiles between the two groups. The metabolic profiles of children with clinical TB compared to microbiologically-confirmed TB were indistinguishable, reflected by the OPLS-DA models’ low or negative predictive ability (Q^2^Y scores) (model values given in Supplementary Table 1, Supplementary statistical analysis methods). Therefore, the clinically diagnosed and bacteriologically-confirmed TB patients were grouped together in further analyses to enable greater analytical power, and are subsequently referred to as the ‘TB disease’ group.

### Metabonomic analysis

PCA models were produced using the data acquired from each of the four analytical platforms to identify contributors to variance in the metabolic profiles amongst all participants. The PCA scores plots are shown in Supplementary Fig. 1.

OPLS modelling identified weight as a factor influencing the metabolic profiles obtained (SI, Table 2) and therefore subsequent OPLS-DA models were adjusted for weight.

### ^1^H NMR characterisation of the TB phenotype

**Figure 1:**
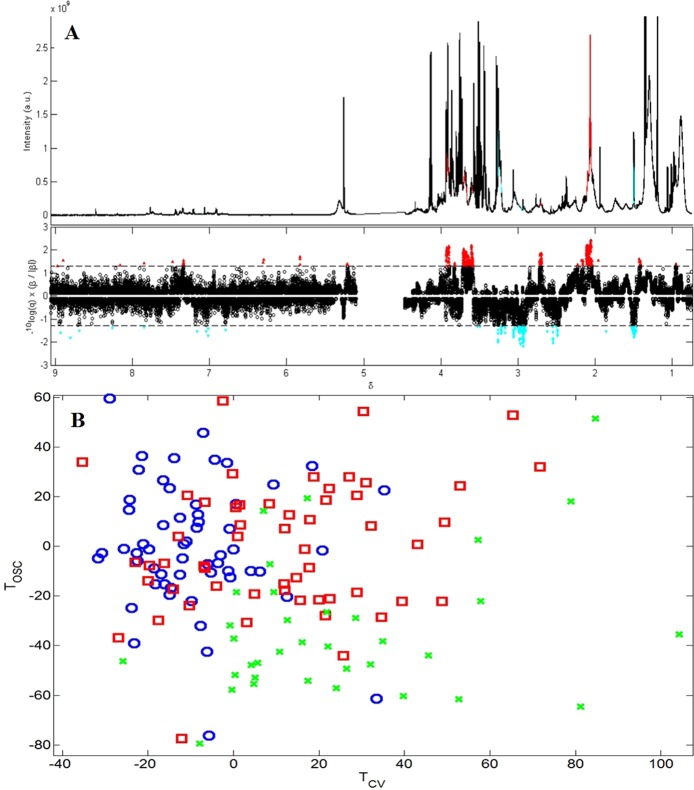
OPLS-DA model based on ^1^H NMR spectroscopy data, with 500 model iterations, separating Gambian infants at enrolment based on diagnosis, R^2^Y = 0.78, Q^2^Y = 0.30, n = 93 (one sample was excluded as there was no information on the participant’s weight, and another was excluded as it was an outlier). (**A**) The upper panel shows the median ^1^H spectra of the serum, with peaks which were statistically significantly different between the two groups highlighted. Peaks in red were found in higher concentrations in the TB disease group, while peaks in blue were found in increased concentrations in the other diseases group. This was plotted for easier identification of the peaks and their corresponding metabolites. The lower panel displays a skyline significance plot of significant variables discriminating between the groups. Variables in red above the dashed line are statistically significantly increased in samples from the TB disease group, the strength of the correlation is displayed by the distance from the dashed line, with variables further away being more strongly associated with that group. Variables in blue below the dashed line are the variables increased in the other diseases group (or found in lower concentrations than in the TB disease group). (**B**) OPLS-DA scores plot, displaying the correlations in the ^1^H spectra between the participants (the closer the scores are the more similar these participants ^1^H NMR spectra are to one another). Red squares represent the TB disease group, blue circles represent the other diseases group, and green crosses represent the validation group samples.T orthogonal signal correction, T_OSC;_ T cross validation, T_CV_.

Figure 1 displays the mean values of a 500 model iteration of the OPLS-DA model comparing the ^1^H NMR metabolic profiles of the TB disease and other diseases groups. Spectral regions discriminating between other diseases and TB disease samples, and corresponding putative metabolite identifications, are shown in Table 2. The OPLS-DA model values are shown in Table 3, and scores plots in Supplementary Fig. 2a (PCA) and 3a (OPLS-DA).

**Table 2:** Chemical shifts discriminating between TB and other diseases in Gambian children at enrolment. Peaks without significant variables on either side were ignored.

	^1^H ppm	Carbon ppm	r (CI lower and upper bound)	P value	Q value FDR	Increased in	^1^H NMR peak multiplicity	Potential metabolite
1	1.427	19.07	0.36 (0.26–0.44)	<0.001	0.03	TB	Doublet	Unknown
2	1.498	19	−0.38 (−0.47– −0.29)	<0.001	0.02	Other diseases	Doublet	Alanine
3	2.044	29.68	0.36 (0.24–0.45)	<0.001	0.03	TB	Multiplet	Glutamate
4	2.054	24.83	0.50 (0.40–0.58)	<0.001	0.004	TB	Broad resonance	N-acetyl glycoprotein (Glyc-A)
5	2.076		0.41 (0.30–0.50)	<0.001	0.01	TB	Broad resonance	O-acetyl glycoprotein
6	2.106	25.14	0.45 (0.36–0.53)	<0.001	0.01	TB	Multiplet	Glutamate
7	2.159		0.36 (0.25–0.44)	<0.001	0.03	TB	Multiplet	Glutamate
8	2.489		−0.37 (−0.50– −0.24)	<0.001	0.02	Other diseases	Triplet	Unknown
9	2.549		−0.41 (−0.51–−0.30)	<0.001	0.01	Other diseases	Doublet	Unknown
10	2.715		0.38 (0.27–0.48)	<0.001	0.02	TB	Multiplet	Glutamate
11	2.931		−0.44 (−0.53–−0.35)	<0.001	0.01	Other diseases	Broad resonance	Unknown
12	2.963		−0.42 (−0.51– −0.33)	<0.001	0.01	Other diseases	Broad resonance	Unknown
13	3.258		−0.41 (−0.51– −0.29)	<0.001	0.01	Other diseases	Undetermined	Unknown
14	3.601		0.44 (0.35–0.52)	<0.001	0.01	TB	Undetermined	Unknown
15	3.629		0.36 (0.26–0.44)	<0.001	0.03	TB	Undetermined	Unknown
16	3.642		0.37 (0.27–0.46)	<0.001	0.02	TB	Undetermined	Unknown
17	3.664		0.38 (0.26–0.48)	<0.001	0.02	TB	Undetermined	Unknown
18	3.685		0.39 (0.28–0.50)	<0.001	0.01	TB	Undetermined	Unknown
19	3.692		0.39 (0.29–0.48)	<0.001	0.01	TB	Doublet of doublets	Unknown
20	3.702		0.40 (0.29–0.49)	<0.001	0.01	TB	Doublet of doublets	Glyc-A
21	3.707		0.40 (0.29–0.49)	<0.001	0.01	TB	Singlet	Leucine
22	3.900	68.94	0.43 (0.34–0.52)	<0.001	0.01	TB	Undetermined	Unknown
23	3.925		0.42 (0.32–0.51)	<0.001	0.01	TB	Undetermined	Glyc-A
24	7.342		0.35 (0.22–0.46)	<0.001	0.03	TB	Doublet	Phenylalanine

**Table 3:** Sensitivity, false positive rate, specificity and false negative rates for each of the analytical platforms employed and 95% CI.

	R^2^Y	Q^2^Y	Sensitivity	False negative rate	Specificity	False positive rate	Threshold	AUC
**TB disease Vs Other diseases**								
^1^H NMR n = 93	0.78	0.30	0.69 (0.56, 0.73)	0.31 (0.27, 0.44)	0.83 (0.73, 0.93)	0.17 (0.07, 0.27)	1.78	0.78
HILIC n = 107	0.49	0.23	0.59 (0.49, 0.67)	0.41 (0.33, 0.51)	0.89 (0.75, 0.92)	0.11 (0.08, 0.25)	3.69	0.76
Lipidomics ESI− n = 112	0.47	0.27	0.58 (0.53, 0.64)	0.42 (0.36, 0.47)	0.89 (0.80, 0.96)	0.11 (0.04, 0.20)	3.28	0.78
Lipidomics ESI+ n = 112	0.48	0.23	0.67 (0.60, 0.71)	0.33 (0.29, 0.40)	0.86 (0.75, 0.93)	0.14 (0.07, 0.25)	0.65	0.78
**Bacteriologically confirmed TB Vs Other diseases**								
^1^H NMR n = 65	0.92	0.29	0.82 (0.59, 0.88)	0.18 (0.12, 0.41)	0.77 (0.59, 0.94)	0.23 (0.06, 0.41)	−0.90	0.81
HILIC n = 77	0.63	0.21	0.76 (0.52, 0.81)	0.24 (0.19, 0.48)	0.68 (0.57, 0.86)	0.32 (0.14, 0.43)	−1.12	0.77
Lipidomics ESI− n = 79	0.66	0.14	0.68 (0.55, 0.77)	0.32 (0.23, 0.45)	0.79 (0.64, 0.95)	0.21 (0.05, 0.36)	−1.65	0.74
Lipidomics ESI+ n = 79	0.68	0.17	0.73 (0.59, 0.82)	0.27 (0.18, 0.41)	0.77 (0.59, 0.91)	0.23 (0.09, 0.41)	−1.47	0.78

TB disease sera showed increased concentrations of glutamate, *N* and *O*-acetyl glycoproteins (GlycA) and phenylalanine, and lower concentrations of alanine, compared to the other diseases group, shown in Fig. 1 and Table 2.

### UPLC-MS characterisation of the TB phenotype

To maximize the metabolic phenotype coverage, three complementary chromatographic separations were utilised (lipidomic profiling method with positive and negative electrospray ionization (ESI) - for the detection of complex lipid species (lipidomics ESI+ and lipidomics ESI−, respectively); and hydrophilic interaction liquid chromatography (HILIC) - for the detection of polar molecules).

**Figure 2:**
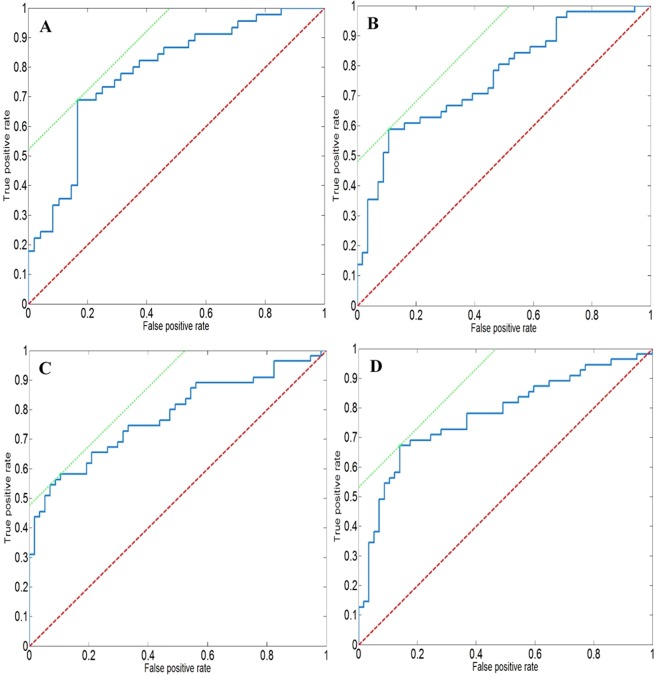
ROC curves displaying the sensitivity and specificity of the OPLS-DA model from data acquired by (**A**) ^1^H NMR spectroscopy, AUC = 0.78 (**B**) HILIC, AUC = 0.76 (**C**) Lipidomics ESI−, AUC = 0.78 (**D**) Lipidomics ESI+, AUC = 0.78. The red line is the line of no-discrimination and the green line gives the slope of the best result.

OPLS-DA models were produced for the UPLC-MS data, comparing the TB disease group against other diseases, to identify systematic distinguishing metabolic variations. Model values are given in Table 3 and Fig. 2, SI. Scores plots for these models are shown in Fig. 3, SI. The OPLS-DA models for all three chromatographic separation methods showed similar predictive abilities.

Lipidomics ESI+ identified ganglioside GM3 (d18:1/16:0), triacylglycerides (16:0/18:1/18:1 and 54:2), and hexose ceramide (d18:1/16:0) as important metabolites distinguishing between the two groups, all of which were increased in the TB disease group relative to other diseases. Lipidomics ESI− showed elevated levels of ceramides (d18:1/16:0, d18:1/20:0, and d18:1/22:0) in the TB disease group. Additional metabolites increased in the TB disease group included lactosylceramide (d18:1/16:0), and HEX-ceramide (d18:1/16:0). Variables identified as distinguishing between the groups are given in Fig. 4, SI, and SI Tables 3–5.

The discriminant features did not share their molecular mass, retention time, or fragmentation patterns with molecules of mycobacterial origin and were therefore presumably of host rather than pathogen origin.

### Analysis of bacteriologically confirmed TB compared with other diseases

To investigate whether comparing only bacteriologically confirmed TB cases against other diseases gave improved predictions of case classification, OPLS-DA models were produced. OPLS-DA model values for this comparison are given in Table 3. These models provide a similar predictive capability as the models including the clinically diagnosed participants in the TB disease group, further justifying our approach to combine the TB disease groups into one.

### Diagnostic discrimination

Figure 2 displays the Receiver Operating Characteristic (ROC) curves for the ^1^H NMR spectroscopy and mass spectrometry data, with sensitivity, specificity and AUC values summarised in Table 3.

The metabolic signature for TB disease acquired using ^1^H NMR data displayed a sensitivity of 69% (95% confidence interval [CI], 56–73%) and a specificity of 83% (95% CI, 73–93%) with an overall AUC of 0.78.

The data acquired using HILIC, lipidomics ESI-, and lipidomics ESI+ displayed respective sensitivities for TB disease of 59% (95% CI, 49–67%) 58% (95% CI, 53–64%) and 67% (95% CI, 60–71%), and specificities of 89% (95% CI, 75–92%), 89% (95% CI, 80–96%) and 86% (95% CI, 75–93%). The AUC for HILIC, lipidomic ESI−, and lipidomic ESI+ data were 0.76, 0.78, and 0.78, respectively.

Table 4 provides sensitivity and specificity values for other diagnostic tests already in routine use for the diagnosis of paediatric TB.

**Table 4:** Diagnostic performances of routine and potential methods for TB in paediatric populations relative to the metabonomic signatures in this study.

Diagnostic Test	Sensitivity (95% CI)	Specificity (95% CI)
AFB Smear microscopy^5^	26% (14–39)	100% (99–100)
Xpert MTB/Rif assay^5^	62% (51–73)	98% (97–99)
Urine lipoarabinomannan (LAM)^54^	48% (38–59)	61% (56–65)
TB-LAMP^55^	78% (71–83)	98% (96–99)
51 transcript RNA expression signature^13^	83% (67–94)	84% (75–93)
T-cell activation marker-TB assay (TAM-TB)^16^	83% (57–96)	97% (89–100)
^1^H NMR spectroscopy	69% (56–73)	83% (73–93)
HILIC	59% (49–67)	89% (75–92)
Lipidomics ESI−	58% (53–64)	89% (80–96)
Lipidomics ESI+	67% (60–71)	86% (75–93)

Confusion matrices for ^1^H NMR, HILIC and lipidomics ESI− mode are shown in SI Tables 6–9.

### UK validation cohort

Data acquired from the Gambian samples were used to predict the TB disease status of a validation cohort of UK samples, which equally included bacteriologically-confirmed and clinically diagnosed TB cases recruited from household contacts ^1^H NMR spectroscopy correctly classified 6/30 (20%) of the validation cohort. HILIC, lipidomics ESI− and lipidomics ESI+ mass spectrometry data correctly classified 15/35 (43%), 20/36 (56%) and 30/36 (83%) of the UK validation cohort, respectively.

Using the model built from data comparing only bacteriologically confirmed TB against the other diseases groups from the Gambian samples, ^1^H NMR spectroscopy was able to correctly classify 26/30 (87%) of the validation cohort. The HILIC, lipidomics ESI− and lipidomics ESI+ mass spectrometry data correctly classified 29/35 (83%), 24/36 (68%) and 30/36 (83%) of the UK validation cohort, respectively.

## Discussion

This study from The Gambia and the UK used metabolic profiling assays applied to the serum of children with presumptive TB to detect and validate novel diagnostic biomarkers and alterations in host metabolism due to TB disease. The most discriminatory MS data showed a sensitivity of 67%, specificity of 85% and correctly classified 83% of the validation cohort.

^1^H NMR spectroscopy has previously been applied to paediatric plasma samples to assess the diagnostic potential in TB^20^. Sun *et al*. also recruited a validation cohort to test the model produced obtaining an AUC of 0.795, a sensitivity of 82.4%, and a specificity of 83.9%^20^, similar to the values we obtained.

Target product profiles have been developed for new TB diagnostics by the WHO^21^. The WHO recommends the sensitivity of a new diagnostic test for pulmonary TB in children to be equal or above 66% for bacteriologically-confirmed TB (equal to the sensitivity of the Xpert MTB/RIF assay), while recommending a specificity of 98% for childhood TB, compared to a microbiological reference standard^21^.

The tests described here hence pass the optimal requirements for the sensitivity for both ^1^NMR spectroscopy and lipidomics ESI+ , but fail for HILIC and lipidomics ESI−. None of the analytical approaches met the WHO requirements for specificity, and therefore would require further development if to be used as a rule-out test. However, within a step-wise screening algorithm, sensitivity would rank higher than the requirement for specificity.

A strength of the current study is the inclusion of symptomatic children with other diseases as a control group, given the real life situation facing health care professionals in the field who have to be able to distinguish between these two entities in the presence of overlapping symptoms. One of the listed characteristics of an ideal paediatric TB biomarker is indeed the ability to discriminate children with TB disease from those infected with other pathogens^22^.

We also detected novel biomarkers in a readily available, non-sputum based biofluid, particularly important for the diagnosis of paediatric TB, due to the difficulty of obtaining respiratory samples from children. Furthermore, the UK validation cohort confirmed that lipidomics ESI+ can detect TB in children from different environmental and genetic backgrounds, supporting this methodology.

Comparing between the metabonomic approaches, analysis using ^1^H NMR spectroscopy provides robust and reproducible data while MS has greater sensitivity. The complementary chromatographic methods used allow the detection of different classes of metabolites, including lipids and hydrophilic metabolites, including those of potentially mycobacterial origin. Detecting bacterial lipids in human tissue has been demonstrated previously^23^, but given the paucibacillary nature of TB in children, it is especially challenging in this context, and we did indeed not identify any compounds of mycobacterial origin.

In this study, phenylalanine was increased in children with TB disease. Phenylalanine levels are known to differ in serum during TB disease, although it has not previously been demonstrated in children. Che *et al*. observed lower levels of phenylalanine, decreasing 2.73 fold in the TB group^24^. However, Weiner *et al*. identified phenylalanine as increasing in relative abundance in their TB group, while a further study by Zhou *et al*. also identified increased concentrations of phenylalanine in TB disease^25^, corroborating our results. Similarly, another study analysing urine samples identified dysregulated phenylalanine metabolism in TB patients, possibly due to altered gut microbiota^26^.

We also identified increased concentrations of glutamate in the serum of children with TB, in line with findings by Zhou *et al*.^25^, and Frediani *et al*.^27^. TB is able to employ glutamate as an alternative carbon source under hypoxic conditions^28^, and Frediani *et al*. hypothesised that the increased concentrations of glutamate observed in TB patients’ blood may result from increased glutamate synthesis by *M.tb* as a sign of host-pathogen metabolic interactions^27^.

Another amino acid, alanine, has previously shown to be increased in paediatric TB disease^20^. However, Zhou *et al*. identified an opposite relationship, with alanine decreasing^25^, as we also describe. This may be a consequence of increased amino acid oxidation in comparison to protein anabolism, which contributes to the wasting associated with TB^29^.

^1^H NMR spectroscopy showed elevated levels of GlycA in the serum of TB patients in our study. This biomarker is associated which chronic inflammation and long-term risk of severe infection, such as septicaemia and pneumonia^30^.

We also identified increased concentrations of several ceramides in the serum of children with TB disease, particularly ceramide (d18:1/16:0), as well as several glycosylated species of ceramide (d18:1/16:0). This finding supports previous findings of increased concentrations of ceramide (d18:1/16:0) in adult TB patients in comparison to healthy control patients, and patients with community-acquired pneumonia^31^. Raised levels of ceramide (d18:1/16:0) have also been reported in patients with TB in comparison to those with lung cancer^32^.

Ceramides are a type of sphingolipid (sphingosine plus a fatty acid), and are present in high concentrations in cell membranes. Ceramides are considered potent bioactive lipids, involved in multiple cellular signalling pathways^33^. Ceramide contributes to phagosome maturation in macrophages infected with *M.tb*, resulting in increased killing of pathogenic mycobacteria^34^. Furthermore, activation of natural killer T cells by the CD1d ligand α-galactosylceramide has been shown to protect mice against TB. Treatment with α-galactosylceramide has been shown to reduce the bacterial burden in the lungs, while diminishing tissue injury and prolonging survival^35^. They are also known to contribute to cellular invasion^36^, apoptosis^37^, and cell-cell signalling^38^, all of which relate intimately to microbial pathogenesis.

Similarly, ganglioside GM3 (d18:1/16:0), another sphingolipid, was increased in children with TB disease in this study. Gangliosides are degraded to ceramides by removal of the sugar units in the oligosaccharide head group, and similarly to ceramides, are present in cell membranes, being particularly concentrated in lipid rafts^39^. The oligosaccharide group found on gangliosides protrude from the cell membrane^40^, and are involved in cell-cell interactions, signal transduction and cell activation. Gangliosides have previously been shown to be involved in *M. leprae* infection^41^.

We hypothesise that ceramides and gangliosides are increased in serum of children with TB disease as a consequence of the immune system’s attempt to kill *M.tb* through the maturation of phagosomes in macrophages, as well as the production of lipid rafts as signalling platforms, to internalise *M.tb*, induce apoptosis and regulate cytokine responses.

Our study has some clear limitations: our sample size was modest, and - as with many paediatric TB studies, a high proportion of cases were diagnosed using a clinical rather than microbiological case definition and there was no blinding for the reading of chest X rays, which nevertheless were read by two independent doctors. Data on nutritional status beyond weight are unfortunately not available as an analytical variable but we showed strong correlation with age. The validation cohort did not include an “other diseases” group, and therefore specificity in this UK population could not be evaluated.

We included symptomatic children in our study to address the “real-world” problem of assessing symptomatic children who have recently been exposed to TB. The model helps to discriminate between TB and other diseases. However, it is unknown if the model would discriminate between children with asymptomatic TB infection and symptomatic disease. This is a subject for a subsequent study.

Additionally, not every laboratory method could be applied to all samples due to limited serum volumes. We are unsure why the model using only bacteriologically confirmed TB from the Gambia was so much better at predicting the disease status of the UK validation cohort than the combination of bacteriologically and clinically diagnosed TB cases. Children in the bacteriologically confirmed cohort in the UK were however older and more likely to be IGRA and TST-positive. We fully acknowledge that further validation will need to be undertaken to confirm the biomarkers identified in this study in a larger population, and in the context of HIV co-infection, as all of the children enrolled in the UK and The Gambia were HIV-negative.

In conclusion, we have demonstrated that alterations in host metabolism in paediatric TB are detectable using metabonomic techniques applied to small volume serum samples. The metabolic profiles provide insights into the metabolic processes associated with TB, but further validation is required to assess the clinical utility of this diagnostic approach in the context of screening algorithms.

## Participants and Methods

Serum samples were obtained from prospectively recruited Gambian children with presumptive TB, who were identified by household TB contact tracing, or referred directly from community health centres, to a dedicated paediatric TB clinic at the MRC Unit The Gambia, as previously described^4,6^.

All children living in the same household as an adult with pulmonary TB in the Greater Banjul area were screened with a symptom questionnaire and tuberculin skin test (TST). Any child with symptoms compatible with TB was referred to the childhood TB clinic for further investigation, including CXR, microbiological investigations, and blood samples collected for immune profiling studies.

The TB disease status of participants was defined in accordance with the case definitions proposed by the World Health Organisation^42^, classified as either bacteriologically-confirmed or clinically diagnosed TB, given that the proposed NIH classification excludes children with a record of household exposure^43^.

The “other diseases” group included participants from the same household cohort whose symptoms were potentially compatible with pulmonary TB but resolved spontaneously, or with short-course conventional antibiotic treatment, and who had no radiological evidence of TB disease, no bacteriological confirmation and did not develop TB disease during the 12 months of regular follow up of the cohort.

Samples from all children consecutively diagnosed with TB disease between February 2012 and June 2014 were included, together with a random selection of children with other diseases form the same setting and investigated during the same time period, at a ratio of 3:1 with TB cases. The selection criteria have been previously described^44^. The Supplementary Information (SI) provides further details on the case definitions used (Supplementary Methods).

Ethics approval was granted by The Gambia Government/Medical Research Council Joint Ethics Committee (ref L2012.E01).

Samples obtained from the UK were part of the NIHR-funded IGRA Kids Study (NIKS), a prospective multicentre collaborative study aiming to assess the negative predictive value of IGRA in children exposed to TB^7,45^. As part of the mandatory TB contact-tracing undertaken according to national guidelines in the UK, all children (<15 years) with a history of household exposure to a source case, presenting to five paediatric TB clinics in London, together with paediatric TB clinics in Southampton, Bristol, Birmingham, Manchester, Glasgow and Newcastle between 1 January 2011 and 31 December 2014 were recruited for screening and investigations. Evaluations included history, examination, TST and IGRA tests, chest radiography, microbiology and HIV testing where appropriate. Samples included from the NIKS study came from consecutively recruited participants with either clinically diagnosed or bacteriologically-confirmed TB. The NIKS study was approved by the National Research Ethics Service (REC: 11/11/11) and cohort details have previously been published All research was performed in accordance with the relevant regulations and informed consent was obtained from the legal guardians of all participants.

### Sample preparation and analysis

Serum was collected at the time of enrolment, separated within 4 hours, aliquoted, and stored at −80 °C prior to shipping on dry ice to Imperial College London, where they were preserved at −80 °C until analysis at the Clinical Phenotyping Centre.

Sample handling and quality control procedures have been reported previously^46^ and details on sample preparation are included in the SI.

All UPLC-MS analyses were performed on Acquity UPLC instruments coupled to Xevo G2-XS oaTOF mass spectrometers (Waters Corp., Manchester, UK) via a Zspray ESI source.

Details of the system configuration and analytical methods used for HILIC profiling have been reported previously^46^, with the exception of the sample preparation procedure, which was modified for application to serum and is reported in the SI.

Lipidomic profiling was conducted using an Acquity 2.1 × 100 mm BEH C8 column thermostated at 55 °C. Solvent A consisted of a 50:25:25 mixture water/acetonitrile/isopropanol with 5 mM ammonium acetate, 0.05% acetic acid, and 20 µM phosphoric acid (which was added to improve the peak shape of some phospholipid species^47^. Solvent B consisted of 50:50 acetonitrile/isopropanol with 5 mM ammonium acetate and 0.05% acetic acid. Initial conditions were 99:1 A:B with a flow rate of 0.6 mL/min. Additional chromatographic and spectrometric conditions for both ion modes can be found in the SI. Sample preparation for lipidomic profiling was performed as described previously^48^, with minor modifications described in the SI.

Significant features identified by lipidomics were compared to the MycoMass database to identify any matches relating to metabolites of potential bacterial origin^49^.

Samples were prepared for ^1^H NMR spectroscopic analysis in accordance with sample preparation protocols previously validated for serum in the section of Computational and Systems Medicine at Imperial College^50^. Further details on the ^1^H NMR spectroscopic analysis can be found in the SI. Due to limited sample volumes, some samples could not be analysed using all four analytical assays.

### Statistical analysis

Unsupervised Principal component analysis (PCA) models, which do not include knowledge of the results of the reference standard, were produced to investigate whether there were any hard outliers, due to either analytical error or biological deviation. PCA is a multivariate projection method, used to extract and display systematic variation in a data matrix. The scores plots of the PCA models display correlations between the participants metabolic profiles, with points closer together representing more similar profiles, allowing groups and trends to be revealed^51^.

Orthogonal partial least squares-discriminant analysis (OPLS-DA) is an extension of PCA, also a multivariate modelling method, used to connect the metabonomic data to the class (diagnosis). It was used to predict the diagnosis of participants, identifying variables that discriminate between classes. OPLS-DA models were run using a Monte Carlo cross-validation strategy to avoid over-training and reliance on a single model^52^, with the average correlations of the projected scores and the data projected onto the spectrum for ^1^H NMR spectrometry data. MS data were treated in the same way.

To account for the influence of bodyweight in the metabolic profiles (Supplementary Table 2), a resampling strategy was implemented during the modelling process of the OPLS-DA models. Using the distribution of the body weights of the TB cases, samples from children with other diseases were sampled with probabilities of each being selected dependent on their weight. Further information on the statistical analysis and metabolite identification can be found in the SI.

## Supplementary information

Supplementary information.
