# Supplementary material for: Performance of metabonomic serum analysis for diagnostics in paediatric tuberculosis
Source: Sci Rep. 2020 Apr 29;10:7302. doi: 10.1038/s41598-020-64413-6 (PMC7190829; doi:10.1038/s41598-020-64413-6)
Supplement: Supplementary file 1 — Supplementary information. [file 41598_2020_64413_MOESM1_ESM.docx]

**Supplementary Information**

Performance of metabonomic serum analysis for diagnostics in paediatric tuberculosis

Nicholas J. Andreas, PhD; Robindra Basu Roy, PhD; Maria Gomez-Romero, PhD; Verena Horneffer-van der Sluis, PhD; Matthew R. Lewis, PhD; Stephane S.M. Camuzeaux BSc; Beatriz Jiménez, PhD; Joram M. Posma, PhD; Leopold Tientcheu, PhD; Uzochukwu Egere, PhD; Abdou Sillah, MSc; Toyin Togun, PhD; Elaine Holmes, PhD and Beate Kampmann, PhD

**Patient characteristics…………………………………………………………………………………………………………………..2**

Table 1: OPLS-DA model values comparing different diagnoses against one another……………………..…2

**Supplementary statistical analysis methods………………………………………………………………………………….2**

Table 2: OPLS model values identifying the influence of weight on the metabolic profiles acquired…………………………………………………………………………………………………………………………………….……...3

**Supplementary figures………………………………………………………………………………………………………………….4**

Figure 1: PCA scores plots…………………………………......………………………………………………………………………...4

Figure 2: Histogram of the distribution of the values of R^2^Y and Q^2^Y of the 500 OPLS-DA model …………………………………………………………………………………………………………………………….…………………………..5

Figure 3: Cross-validated scores plots of the corrected OPLS-DA models…………………………………..……..6

Figure 4: Variables which are significantly correlated with tuberculosis cases, and other disease samples……………………………………………………………………………………………………………………………………….……7

**Supplementary tables………………………………………………………………………………………………………………….9**

Table 3: Most discriminatory variable selected by HILIC…………………………………………………………………..9

Table 4: Most discriminatory variables selected by Lipidomics ESI+…………………………………………………9

Table 5: Most discriminatory variables selected by Lipidomics ESI-………………………………………………….14

Table 6: Confusion matrix based on the cross-validated model from ^1^H NMR spectroscopy data…….20

Table 7: Confusion matrix based on the cross-validated model using data acquired by HILIC………....20

Table 8: Confusion matrix based on the cross-validated model using data acquired by Lipidomics ESI-……………………………………………………………………………………………………………………………………………..……20

Table 9: Confusion matrix based on the cross-validated model using data acquired by Lipidomics ESI+…………………………………………………………………………………………………………………………………………..……20

**Supplementary methods…………………………………………………………………………………………………………….21**

Case definitions…………………………………………………………………………………………………………………………..….21

Mass spectrometry analysis……………………………………………………………………………………..…………………….21

Sample sorting, formatting, and aliquoting……………………………………………………………………………….…....21

Study reference (SR), and SR dilution ………………………………………………………………..………….……………….21

*Table 10. HILIC MR standards*……………………………………………………………..…………………………...22

Sample preparation for UPLC-MS analysis ………………………………………………………….…………………………22

*Table 11. Standard mixtures for HILIC and lipidomics*……………………………………………………..23

Chromatographic conditions……………………………………………………………………………………….………………….23

Mass spectrometry………………………………………………………………………………………………….……….…………….24

^1^H NMR spectroscopic analysis…………………………………………….…………………………………………….………….24

**Metabolite Identification……………………....……………………………………………………………………..……………24**

**References…………………………………………………………………………………………………………………………….….…25**

# Patient characteristics

Table 1: SIMCA OPLS-DA model values comparing different diagnoses against one another, and analytical platform used. All models computed using two components, produced in SIMCA.

|  | **R^2^X** | **R^2^Y** | **Q^2^Y** | **N** | **Permutation test intercept** |
| --- | --- | --- | --- | --- | --- |
| **^1^H NMR spectroscopy** | | | | | |
| Other diseases vs confirmed TB | 0.09 | 0.85 | 0.15 | 66 | -0.20 |
| Other diseases vs clinically diagnosed TB | 0.10 | 0.76 | 0.15 | 78 | -0.18 |
| Clinically diagnosed vs confirmed TB | 0.12 | 0.88 | 0.06 | 46 | - |
| **HILIC ESI+** | | | | | |
| Other diseases vs confirmed TB | 0.28 | 0.39 | 0.02 | 78 | - |
| Other diseases vs clinically diagnosed TB | 0.2 | 0.4 | 0.06 | 87 | - |
| Clinically diagnosed vs confirmed TB | 0.27 | 0.42 | -0.29 | 51 | - |
| **Lipidomics ESI-** | | | | | |
| Other diseases vs confirmed TB | 0.3 | 0.45 | 0.00 | 79 | - |
| Other diseases vs clinically diagnosed TB | 0.29 | 0.42 | 0.12 | 90 | -0.25 |
| Clinically diagnosed vs confirmed TB | 0.26 | 0.37 | -0.13 | 55 | - |
| **Lipidomics ESI+** | | | | | |
| Other diseases vs confirmed TB | 0.38 | 0.38 | 0.04 | 79 | - |
| Other diseases vs clinically diagnosed TB | 0.35 | 0.37 | 0.14 | 90 | -0.26 |
| Clinically diagnosed vs confirmed TB | 0.3 | 0.32 | -0.14 | 55 | - |

# Statistical analysis

R^2^X denotes the fraction of the variation of the X variables (the spectroscopic/spectrometric data) explained by the model, i.e. it describes how well the model fits the data, with 1.00 denoting a perfect model (no noise in the data), in PCA models of biological data, a R^2^ score of around 0.5 can be expected.

R^2^Y denotes the fraction of the variation of the Y variables (the class information, e.g. age/or diagnosis) that is explained by the model, with 1.00 being the best model possible^1^.

Q^2^ is an estimate of the predictive ability of the model. It is calculated by cross-validation. The data are divided into 7 parts and each 1/7th in turn is removed. A model is built on the 6/7th data left in and the left out data are predicted from the new model. This is repeated with each 1/7th of the data until all the data have been predicted. The predicted data are then compared with the original data and the sum of squared errors calculated for the whole dataset. This is then called the Predicted Residual Sum of Squares (PRESS). The better the predictability of the model the lower this value will be. For convenience we then convert PRESS into Q^2^ to resemble the scale of the R^2^. PRESS is divided by the initial sum of squares and subtracted from 1. Good predictions will have low PRESS and so high Q^2^.’’ ^2^ A Q^2^ score of 1 would indicate a perfectly predictive model. For data relating to biological models, a Q^2^ of approximately 0.4 can be expected ^2^.

OPLS-DA models incorporate data on the results of the reference standard.

**Table 2:** OPLS model values identifying the influence of weight on the metabolic profiles acquired, and analytical platform used. All models computed using two components, produced in SIMCA.

|  | **R^2^X** | **R^2^Y** | **Q^2^Y** | **N** | **Permutation test intercept (100 permutations)** |
| --- | --- | --- | --- | --- | --- |
| **^1^H NMR spectroscopy** | 0.10 | 0.86 | 0.15 | 94 | -0.14 |
| **HILIC ESI+** | 0.29 | 0.63 | 0.31 | 108 | -0.28 |
| **Lipidomics ESI-** | 0.16 | 0.64 | 0.36 | 111 | -0.35 |
| **Lipidomics ESI+** | 0.21 | 0.49 | 0.17 | 111 | -0.32 |

^1^H NMR spectra were imported into Matlab R2014a (MathWorks Inc. USA), referencing the chemical shifts to Trimethylsilylpropanoic acid (TSP). Water and TSP signals were subsequently removed from the spectra. NMR spectra were normalised using probabilistic quotient normalisation (median fold change)^3^. ^1^H NMR spectra and mass spectrometry data were scaled using unit variance (UV) scaling ^1^.

Chromatograms were visualised using MassLynx (version 4.1, Waters). Data processing was undertaken using Progenesis QI (Waters) including matching peak picking, retention time correction (alignment), filtering and normalisation.

Matlab R2014a and SIMCA P+ (version 14.0) were used for multivariate data analysis, using principal component analysis (PCA) and orthogonal partial least squares-discriminant analysis (OPLS-DA) to evaluate the metabolic profiles and for biomarker identification. Receiver Operating Characteristic (ROC) curves were calculated using an in-house code in Matlab R2014a.

# Supplementary Figures

**A**

**B**

**C**

**D**

Figure 1: PCA scores plots of (**A**) ^1^H NMR spectroscopy, R^2^X=0.17, Q^2^Y=0.11, n=93 (**B**) HILIC, R^2^X=0.36, Q^2^Y=0.31, n=108 (**C**) Lipidomics ESI-, R^2^X=0.30, Q^2^Y=0.26, n=112 (**D**) Lipidomics ESI+, R^2^X=0.28, Q^2^Y=0.23, n=112. Red are other diseases samples, orange are clinically diagnosed samples and green are confirmed tuberculosis samples.

In PCA the class of the sample (in this case diagnosis) is blinded, so we would not expect to see discrimination between the two groups as there are bigger influences on the participants metabolic profiles than their disease status. This is unsurprising as these are free-living individuals with different genetic profiles and diets. Separation would only be expected in a tightly controlled experiment e.g. animal studies.


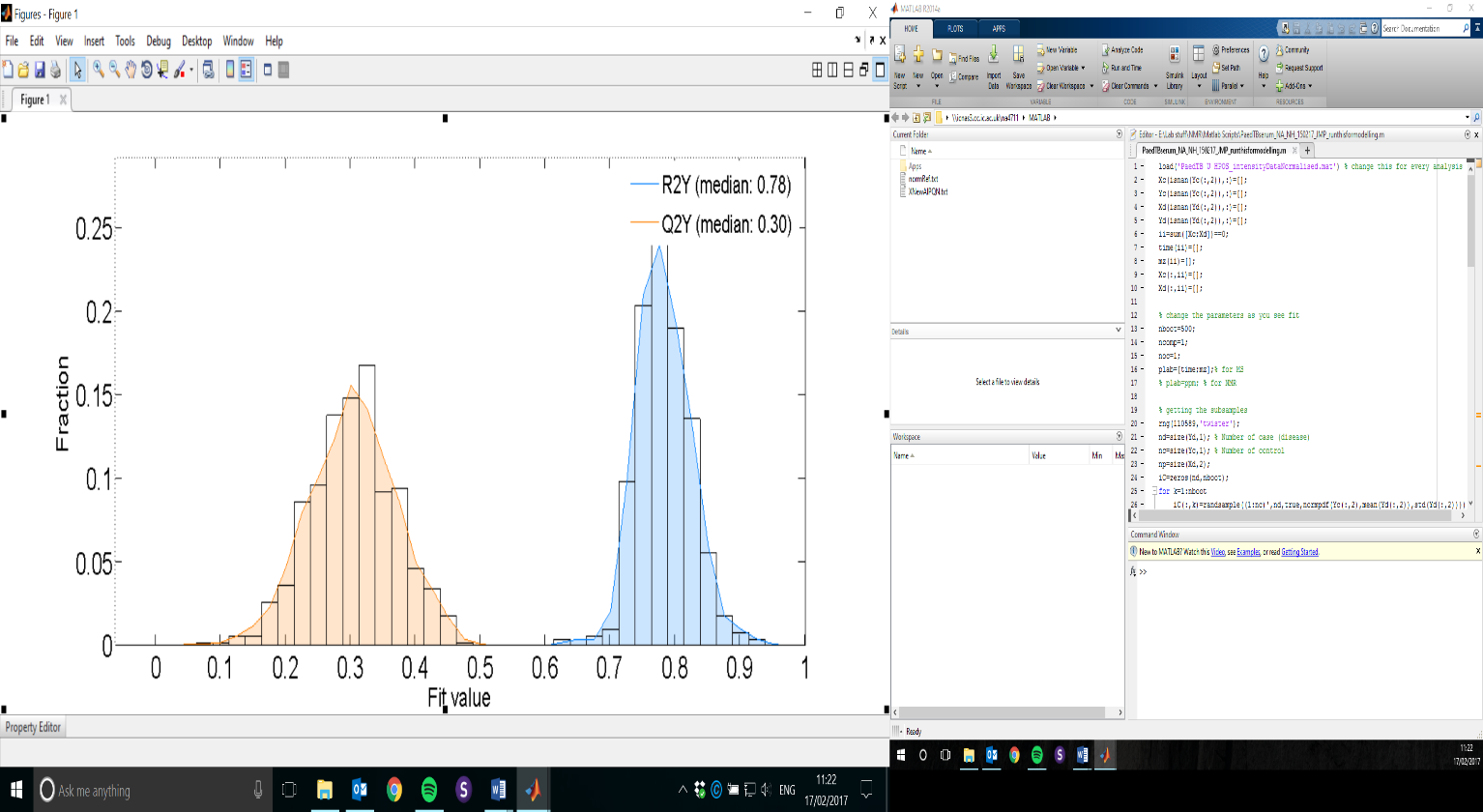

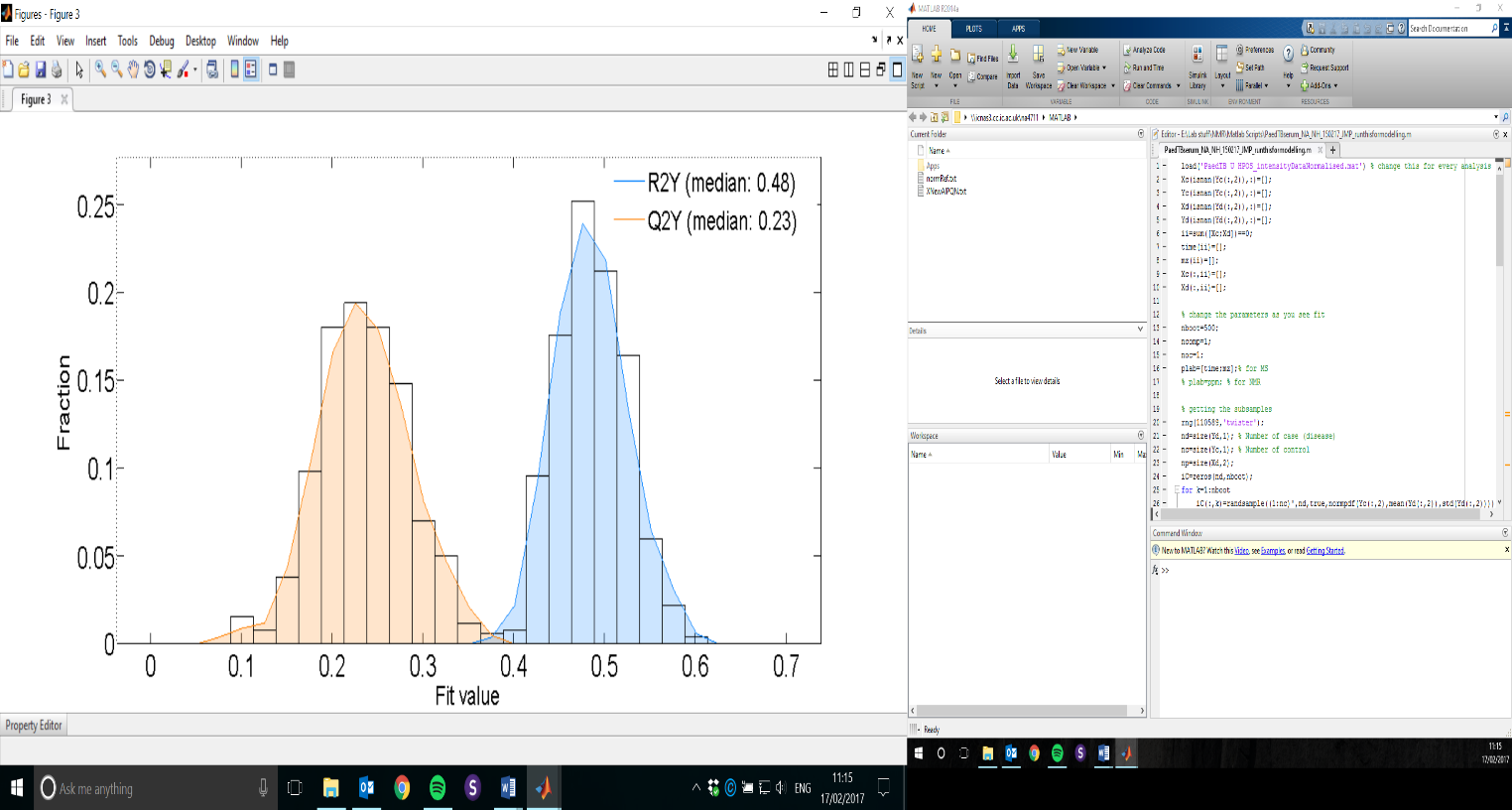

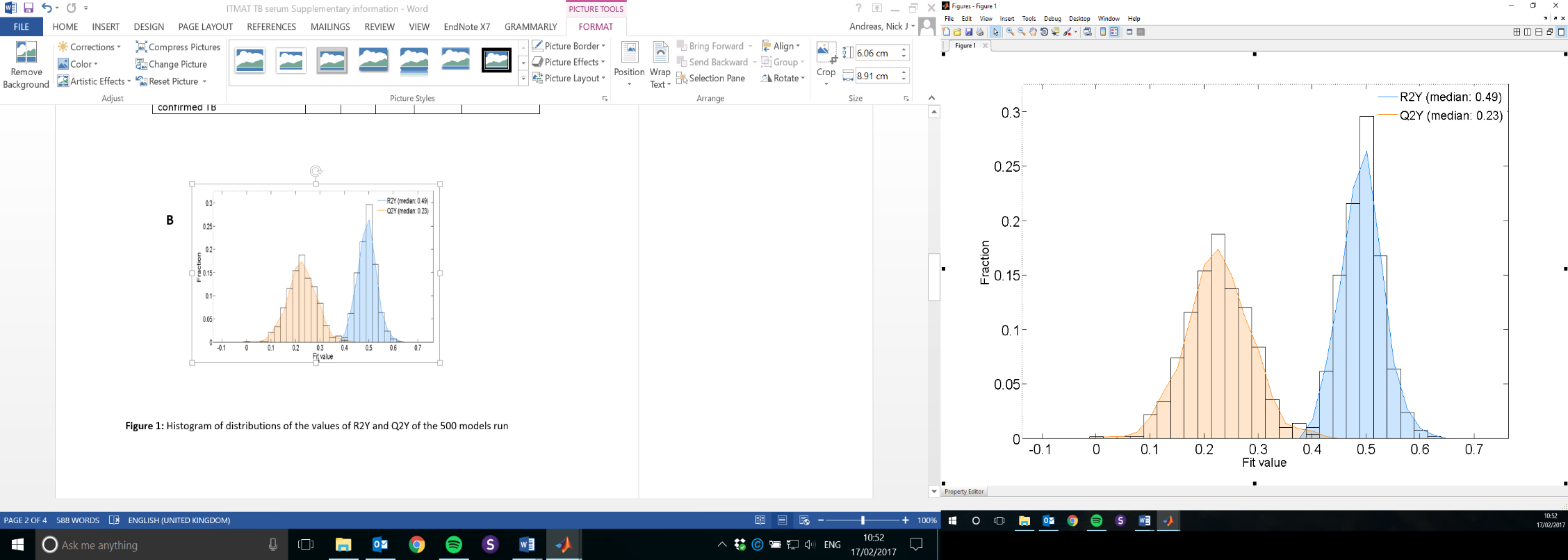


**B**


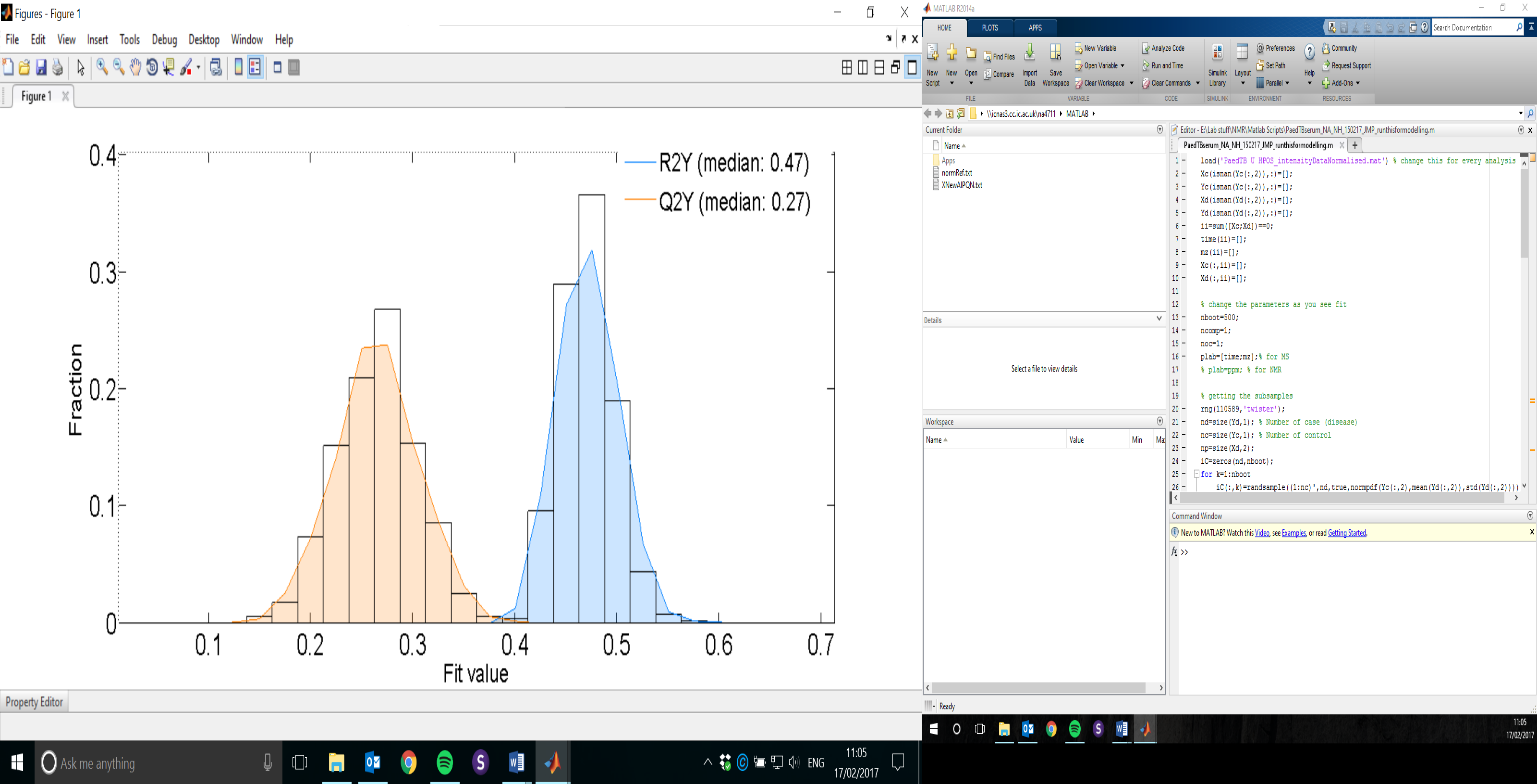


**C**

**D**

**A**

Figure 2: Histogram of the distribution of the values of R^2^Y and Q^2^Y of the 500 OPLS-DA models run, median R^2^Y (blue) and Q^2^Y (orange) values are reported. (**A**) ^1^H NMR spectroscopy, R^2^Y=0.78, Q^2^Y=0.30 (**B**) HILIC, R^2^Y=0.49, Q^2^Y=0.23 (**C**) Lipidomics ESI-, R^2^Y=0.47, Q^2^Y=0.27 (**D**) Lipidomics ESI+, R^2^Y=0.48, Q^2^Y=0.23.

As weight was identified as a factor which influenced the metabolic profiles we ran a 500 model iteration of the OPLS-DA model separating the TB disease and other diseases group, to control for weight. Figure 2 displays histograms of the values of R^2^Y and Q^2^Y for the 500 models comparing TB disease and other diseases groups, corresponding to the models produced in Figure 3 SI.


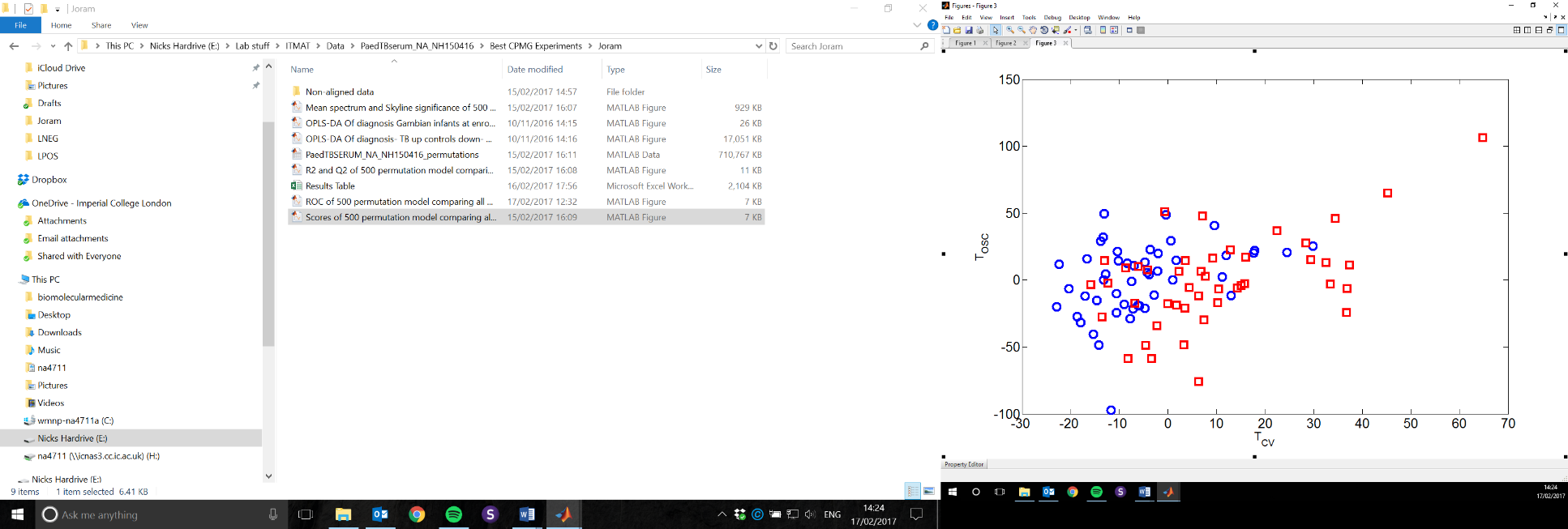

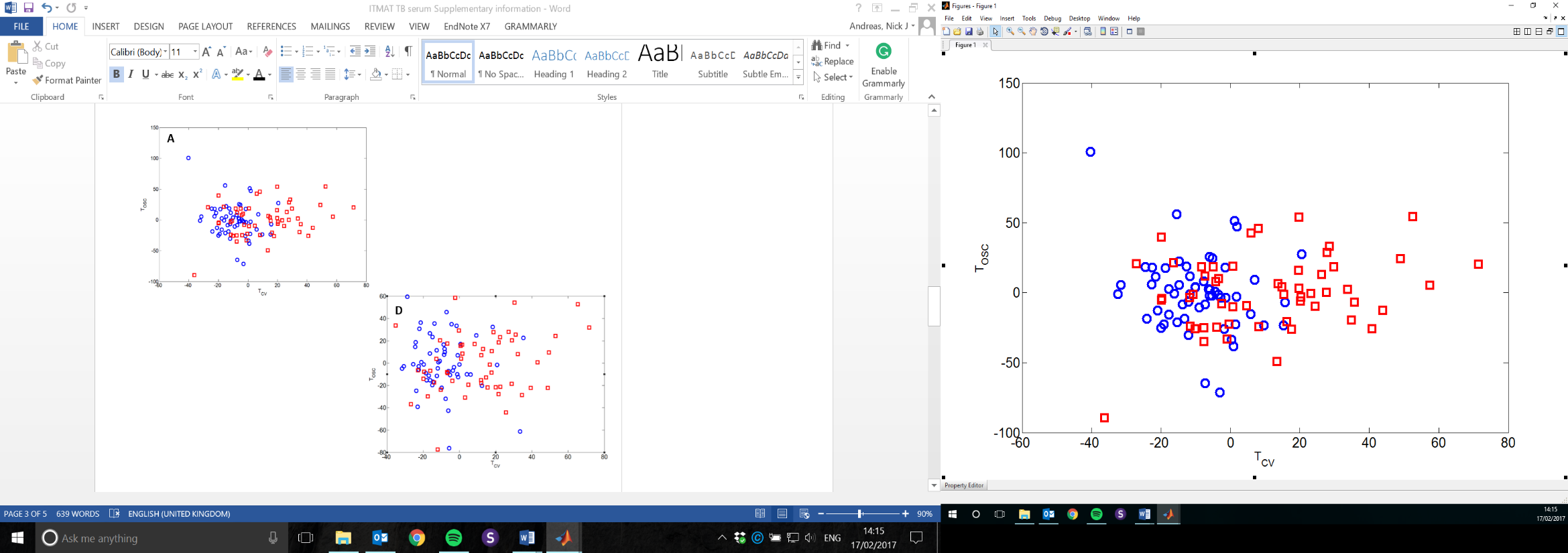

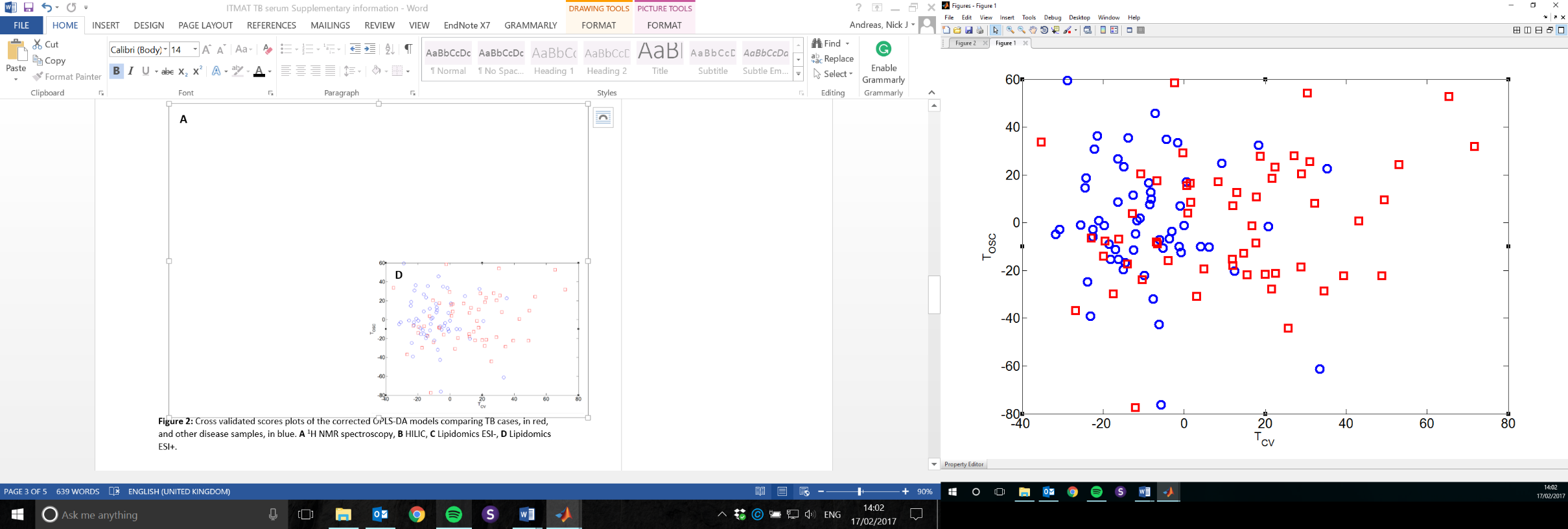


**A**

**D**


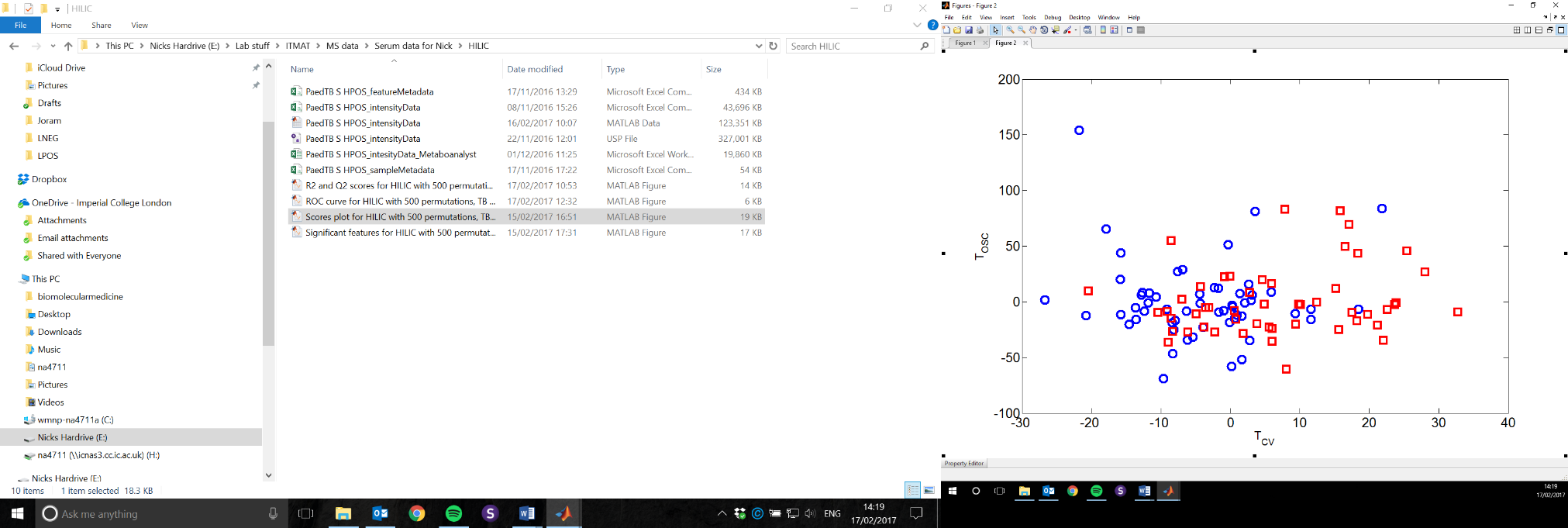


**C**

**B**

Figure 3: Cross-validated scores plots of the corrected OPLS-DA models comparing tuberculosis cases, in red, and other disease samples, in blue. (**A**) ^1^H NMR spectroscopy, (**B**) HILIC, (**C**) Lipidomics ESI-, (**D**) Lipidomics ESI+.

Figure 3 plots the cross validated complement to the regular scores plot, investigating the stability of single points, as well as groups. Firstly 1/7^th^ of the data is kept out of the model, data which is kept out is then predicted by the model, this is then compared with the actual values. This process is repeated until all the data has been kept out of the model once. The cross validated (CV) scores plots resemble the non-CV scores plots which gives confidence in the validity of the model. Also, separation of the groups (the different diagnoses) is seen.


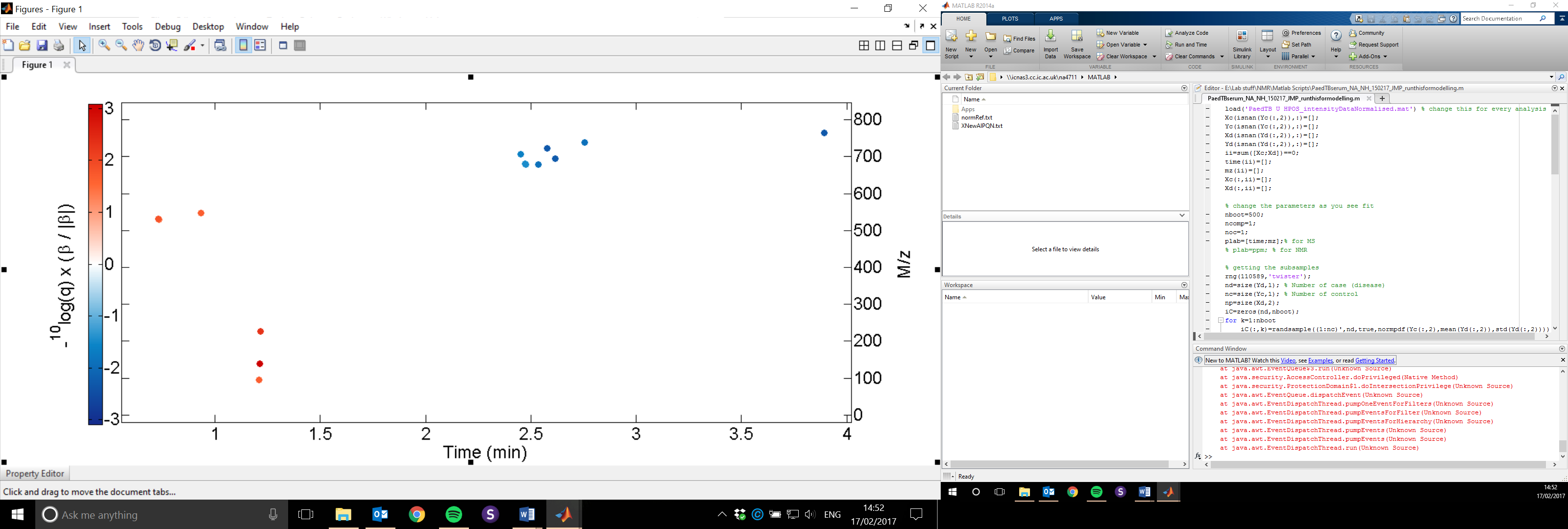

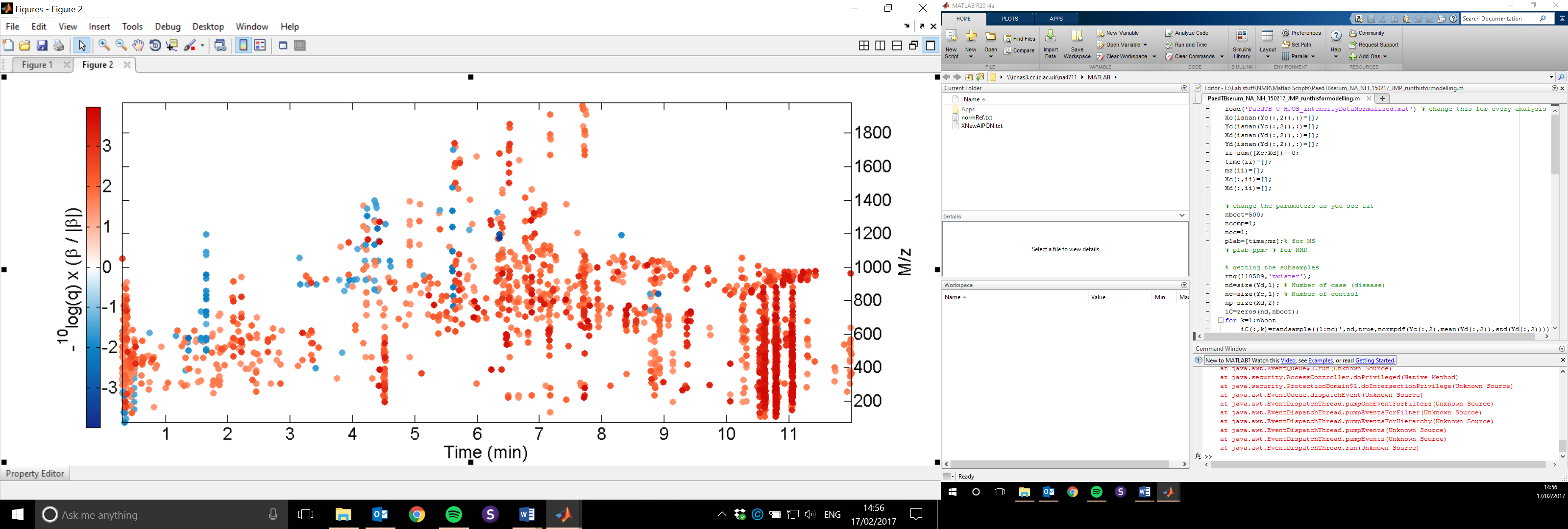


**B**


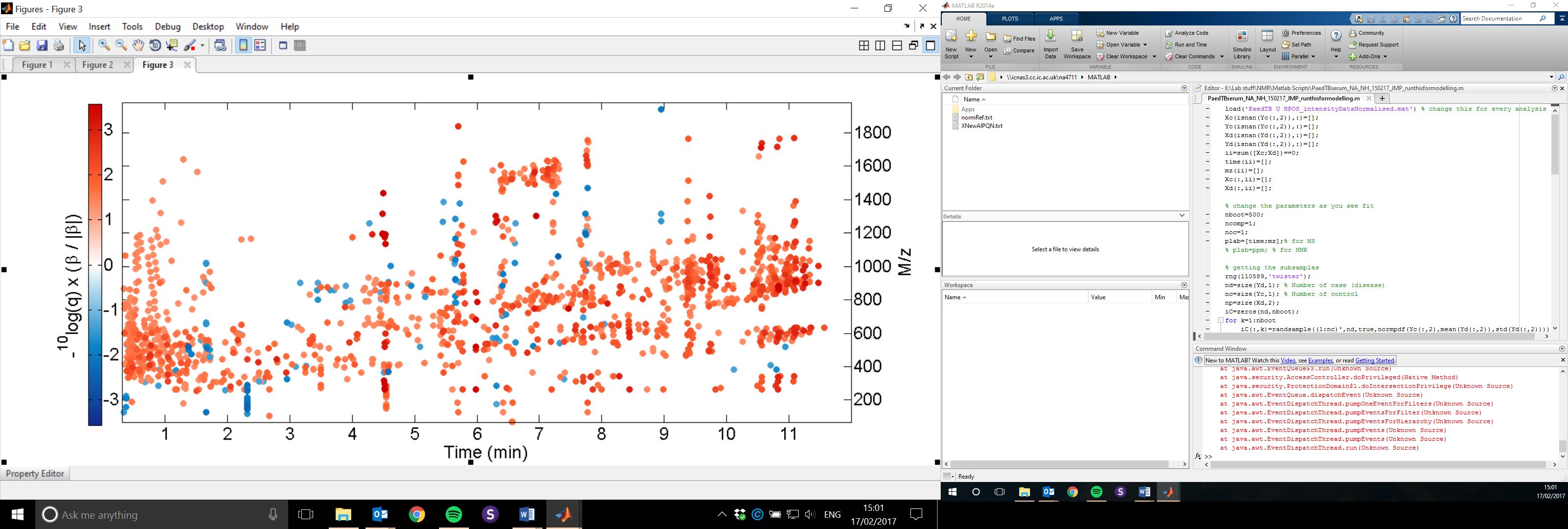


**C**

**A**

Figure 4: Variables which are significantly correlated with tuberculosis cases, in red, and other disease samples, in blue. Variables are selected from the corrected OPLS-DA models. (**A**) HILIC (**B**) Lipidomics ESI- (**C**) Lipidomics ESI+.

Figure 4 displays variables which are significantly associated with disease status for the mass spectrometry data. Along the X axis is time, denoting when the metabolite eluted from the chromatographic column. On the Y axis is m/z ratio, denoting that particular metabolites mass to charge ratio, as well as the strength of the correlation seen between the specific metabolites and the disease status.

# Supplementary Tables

Table 3: Most discriminatory variable selected by HILIC

| ***m/z*** | **Retention time** | **Increased in** | **r**  **(95% CI)** | **P value** | **Q value FDR** | **Comment** |
| --- | --- | --- | --- | --- | --- | --- |
| 138.0554 | 1.212 | TB | 0.48  (0.39—0.55) | 1.84E-07 | 0.000816 | Further MSMS experiment required to allow putative annotation |

Table 4: Most discriminatory variables selected by Lipidomics ESI+, all variables were increased in the TB group.

|  | **Molecular mass *(n)*** | ***m/z*** | **Neutral mass** | **Retention time** | **r**  **(95% CI)** | **P value** | **Q value FDR** | **Adducts** | **Metabolite** | **Formula** | **Comment** |
| --- | --- | --- | --- | --- | --- | --- | --- | --- | --- | --- | --- |
| 1 | - | 1191.67004 | 1152.713 | 4.527 | 0.49  (0.43—0.55) | 5.13E-08 | 0.000273 |  | Ganglioside GM3 (d18:1/16:0),  LEVEL 4A | C_57_H_104_N_2_O_21_ | [M+K]+, based on correlation of MS data |
| 2 | - | 264.26851 | 1152.713 | 4.519 | 0.47  (0.38—0.55) | 2.49E-07 | 0.000289 |  | Ganglioside GM3 (d18:1/16:0),  LEVEL 4A | C_57_H_104_N_2_O_21_ | Fragment of Ganglioside GM3 (d18:1/16:0), based on correlation of MS data |
| 3 | - | 877.03131 | 858.7676 | 10.833 | 0.47  ((0.39—0.53) | 2.58E-07 | 0.000289 |  | TG(52:2) (TG(16:0_18:1_18:1)), LEVEL 2A | C_55_H_102_O_6_ | Ringing of [M+NH4]+ (progenesis), based on MSMS data |
| 4 | 537.5119 | 520.50860 | 699.5649 | 5.985 | 0.47  (0.36—0.56) | 2.75E-07 | 0.000289 | M+H-H2O, M+H | HexCer(d18:1/16:0), LEVEL 4A | C_40_H_77_NO_8_ | Ceramide (d18:1) fragment, based on MS and MSMS data |
| 5 | - | 1437.60212 |  | 4.493 | 0.46  (0.40—0.53) | 3.43E-07 | 0.000289 |  | UNKNOWN |  | Impossible to know ion type, no MSMS, no correlation |
| 6 | - | 264.26846 | 699.5649 | 5.985 | 0.46  (0.35—0.55) | 3.89E-07 | 0.000289 |  | HexCer(d18:1/16:0), LEVEL 4A | C_40_H_77_NO_8_ | Ceramide without the hexose fragment, based on MS and MSMS data |
| 7 | - | 748.62159 |  | 7.471 | 0.46  (0.36—0.53) | 5.74E-07 | 0.000311 |  | UNKNOWN |  | [M+H]+, no correlation at all, MSMS data do not allow annotation |
| 8 | - | 314.08471 |  | 4.512 | 0.45  (0.35—0.53) | 6.40E-07 | 0.000311 |  | UNKNOWN |  | No correlation, the feature is [M+Na]+, no MSMS, several entries in METLIN |
| 9 | - | 1197.68337 |  | 4.489 | 0.45  (0.38—0.52) | 6.41E-07 | 0.000311 |  |  |  | [M+K]+ (could be ganglioside GM3 isomer) |
| 10 | 858.7718 | 881.75599 | 858.7676 | 10.829 | 0.45  (0.36—0.52) | 7.12E-07 | 0.000316 | M+H, M+Na, M+K, 2M+Na | TG(52:2) (TG(16:0_18:1_18:1)), LEVEL 2A | C_55_H_102_O_6_ | Fragment of TG(52:2), based on MSMS data |
| 11 | - | 274.09235 | 1152.713 | 4.516 | 0.45  (0.38—0.51) | 9.22E-07 | 0.000355 |  | Ganglioside GM3 (d18:1/16:0),  LEVEL 4A | C_57_H_104_N_2_O_21_ | Fragment of Ganglioside GM3 (d18:1/16:0), based on correlation of MS data |
| 12 | - | 1175.70214 | 1152.713 | 4.523 | 0.45  (0.37—0.52) | 9.32E-07 | 0.000355 |  | Ganglioside GM3 (d18:1/16:0),  LEVEL 4A | C_57_H_104_N_2_O_21_ | [M+Na]+, based on correlation of MS data |
| 13 | - | 292.10296 | 1152.713 | 4.516 | 0.44  (0.37—0.51) | 1.14E-06 | 0.000369 |  | Ganglioside GM3 (d18:1/16:0),  LEVEL 4A | C_57_H_104_N_2_O_21_ | Fragment of Ganglioside GM3 (d18:1/16:0), based on correlation of MS data |
| 14 | - | 880.92714 | 858.7676 | 10.829 | 0.44  (0.37—0.53) | 1.14E-06 | 0.000369 |  | TG(52:2) (TG(16:0_18:1_18:1)), LEVEL 2A | C_55_H_102_O_6_ | Ringing of [M+H]+ ( progenesis), based on MSMS data |
| 15 | 1152.712 | 1135.70877 | 1152.713 | 4.527 | 0.44  (0.36—0.52) | 1.18E-06 | 0.000369 | M+H-H2O, M+H | Ganglioside GM3 (d18:1/16:0),  LEVEL 4A | C_57_H_104_N_2_O_21_ | [M+H]+, based on correlation of MS data |
| 16 | - | 1317.64223 |  | 4.489 | 0.44  (0.36—0.51 | 1.56E-06 | 0.00045 |  | UNKNOWN |  | Impossible to know ion type, no MSMS, no correlation |
| 17 | - | 881.99479 | 858.7676 | 10.833 | 0.44  (0.34—0.52) | 1.63E-06 | 0.00045 |  | TG(52:2) (TG(16:0_18:1_18:1)), LEVEL 2A | C_55_H_102_O_6_ | [M+Na]+, based on MSMS data |
| 18 | - | 859.87815 | 858.7676 | 10.833 | 0.44  (0.37—0.50) | 1.69E-06 | 0.00045 |  | TG(52:2) (TG(16:0_18:1_18:1)), LEVEL 2A | C_55_H_102_O_6_ | Ringing of [M+Na]+ (progenesis) |
| 19 | - | 1191.66830 |  | 4.489 | 0.44  (0.34—0.52) | 1.94E-06 | 0.000482 |  | UNKNOWN |  | Impossible to know ion type, no MSMS, no correlation |
| 20 | - | 1275.79053 |  | 6.317 | 0.44  (0.38—0.49) | 1.99E-06 | 0.000482 |  | UNKNOWN |  | [M+Na]+, no correlation, no DBs entry, no MSMS |
| 21 | - | 1301.80769 |  | 6.302 | 0.43  (0.37—0.48) | 2.35E-06 | 0.000514 |  | UNKNOWN |  | [M+Na]+ of feature # 465 6.30_1261.8163m/z, no correlation, no DBs entry, no MSMS |
| 22 | - | 1710.48143 |  | 10.551 | 0.43  (0.34—0.51) | 2.42E-06 | 0.000514 |  | UNKNOWN |  | Impossible to know ion type, no entry in DBs, no correlation |
| 23 | 699.5647 | 682.56146 | 699.5649 | 5.985 | 0.43  (0.32—0.53) | 2.50E-06 | 0.000514 | M+H-H2O, M+Na | HexCer(d18:1/16:0), LEVEL 4A | C_40_H_77_NO_8_ | [M-OH]+, based on MS and MSMS data |
| 24 | - | 787.67273 |  | 6.001 | 0.43  (0.31—0.52) | 2.51E-06 | 0.000514 |  | UNKNOWN |  | Impossible to know ion type, no correlation, no MSMS |
| 25 | - | 896.87579 |  | 11.259 | 0.43  (0.37—0.49) | 2.76E-06 | 0.000545 |  | UNKNOWN |  | [M-OH]+, no entry in 3 DB/ no MSMS, no exploitable correlation, correlates with feature #2456 11.26_1001.9913m/z |
| 26 | 537.5111 | 538.51666 | 1152.713 | 4.516 | 0.42  (0.35—0.49) | 4.20E-06 | 0.00075 | M+H-2H_2_O, M+H | Ganglioside GM3 (d18:1/16:0),  LEVEL 4A | C_57_H_104_N_2_O_21_ | Fragment of Ganglioside GM3 (d18:1/16:0), based on correlation of MS data |
| 27 | - | 776.64880 |  | 8.087 | 0.42  (0.34—0.50) | 4.22E-06 | 0.00075 |  | UNKNOWN |  | Impossible to know ion type, no correlation, no MSMS |
| 28 | - | 975.97563 |  | 11.229 | 0.42  (0.35—0.48) | 4.66E-06 | 0.0008 |  | UNKNOWN |  | Only entry in Lipidmaps (none in Metlin, HMDB), no MSMS, correlation do not allow annotation |
| 29 | - | 520.50878 | 1152.713 | 4.516 | 0.42  (0.33—0.50 | 5.47E-06 | 0.000895 |  | Ganglioside GM3 (d18:1/16:0),  LEVEL 4A | C_57_H_104_N_2_O_21_ | Fragment of Ganglioside GM3 (d18:1/16:0), based on correlation of MS data |
| 30 | - | 1001.99133 |  | 11.259 | 0.42  (0.34—0.48) | 5.60E-06 | 0.000895 |  | UNKNOWN |  | No MSMS, no exploitable correlation, correlated with feature #2458 11.26_896.8758m/z |
| 31 | - | 899.89362 |  | 11.467 | 0.42  (0.35—0.48) | 5.71E-06 | 0.000895 |  | UNKNOWN |  | No MSMS, progenesis took the wrong isotope, real m/z is [M-OH]+, no entry in DBs, no exploitable correlation |
| 32 | - | 443.31371 |  | 2.789 | 0.42  (0.33—0.50) | 6.43E-06 | 0.000979 |  | UNKNOWN |  | [M+Na]+, no correlation, several DB entries |
| 33 | - | 1261.81631 |  | 6.298 | 0.41  (0.32—0.48) | 6.64E-06 | 0.000983 |  | UNKNOWN |  | [M-OH]+ of feature # 474 6.30_1301.8077m/z, no correlation, no DBs entry, no MSMS |
| 34 | - | 577.51936 | 858.7676 | 10.829 | 0.41  (0.33—0.48) | 6.89E-06 | 0.000993 |  | TG(52:2) (TG(16:0_18:1_18:1)), LEVEL 2A | C_55_H_102_O_6_ | [M+NH4]+, based on MSMS data |
| 35 | - | 341.30362 |  | 8.276 | 0.41  (0.35—0.48) | 7.35E-06 | 0.001 |  | UNKNOWN |  | Correlation with m/z 605.552, no exploitable correlation for m/z 605.552, could be DG/TG fragment |
| 36 | - | 746.60508 |  | 6.927 | 0.41  (0.34—0.48) | 7.49E-06 | 0.001 |  | UNKNOWN |  | MSMS does not allow us to decipher the annotation (PC and PE with same m/z co-eluting) |
| 37 | 865.8794 | 904.83202 | 886.7989 | 11.096 | 0.41  (0.33—0.48) | 7.51E-06 | 0.001 | M+Na, M+K | TG(54:2) (mainly TG(16:0_18:1_18:1), LEVEL 4A | C_57_H_106_O_6_ | Progenesis miscalculated the ion type/feature, annotation done on main ion, based on MSMS data |

Table 5: Most discriminatory variables selected by Lipidomics ESI-, a positive r value indicates the metabolite is increased in the all tuberculosis group, negative, other diseases.

|  | **Molecular mass *(n)*** | ***m/z*** | **Retention time** | **r**  **(95% CI)** | **P value** | **Q value FDR** | **Adducts** | **Metabolite** | **Formula** | **Comment** |
| --- | --- | --- | --- | --- | --- | --- | --- | --- | --- | --- |
| 1 |  | 523.178 | 0.52 | 0.38  (0.34—0.41) | 5.10E-05 | 0.000527612 |  | UNKNOWN |  |  |
| 2 |  | 425.2003 | 0.52 | 0.37  (0.33—0.41) | 7.11E-05 | 0.000654895 |  | UNKNOWN |  |  |
| 3 |  | 607.4218 | 3.2 | 0.38  (0.29—0.45) | 4.33E-05 | 0.000478243 |  | UNKNOWN |  |  |
| 4 |  | 1167.6967 | 4.25 | 0.39  (0.35—0.42) | 2.95E-05 | 0.000383742 |  | UNKNOWN |  |  |
| 5 |  | 290.0872 | 4.43 | 0.41  (0.32—0.49) | 1.04E-05 | 0.000233031 |  | UNKNOWN |  |  |
| 6 |  | 1151.7035 | 4.43 | 0.43  (0.36—0.5) | 2.40E-06 | 0.000182467 |  | UNKNOWN |  |  |
| 7 |  | 388.0638 | 4.43 | 0.41  (0.33—0.49) | 7.07E-06 | 0.000212328 |  | UNKNOWN |  |  |
| 8 |  | 1271.6603 | 4.44 | 0.4  (0.31—0.48) | 1.37E-05 | 0.000251247 |  | UNKNOWN |  |  |
| 9 |  | 545.2875 | 4.51 | 0.38  (0.28—0.47) | 3.32E-05 | 0.000406759 |  | UNKNOWN |  |  |
| 10 |  | 196.0244 | 4.51 | 0.38  (0.26—0.48) | 3.72E-05 | 0.000443166 |  | UNKNOWN |  |  |
| 11 | 270.2176 | 269.2103 | 4.51 | 0.37  (0.27—0.46) | 8.61E-05 | 0.000739591 | M-H, M+K-2H | UNKNOWN |  |  |
| 12 |  | 459.3283 | 4.52 | 0.39  (0.29—0.49) | 2.30E-05 | 0.000334716 |  | UNKNOWN |  |  |
| 13 |  | 832.5681 | 5.23 | 0.39  (0.27—0.48) | 3.11E-05 | 0.000393007 |  | UNKNOWN |  |  |
| 14 |  | 773.5319 | 5.31 | 0.4  (0.33—0.47) | 1.23E-05 | 0.000247349 |  | UNKNOWN |  |  |
| 15 | 861.6177 | 958.5852 | 5.71 | 0.37  (0.29—0.45) | 6.29E-05 | 0.000602778 | [M+PO_4_H_2_]- | Lactosyl Ceramide (d18:1/16:0), Level 2A | C_46_H_87_NO_13_ |  |
| 16 | 861.6177 | 860.6081 | 5.71 | 0.37  (0.29—0.44) | 6.63E-05 | 0.000624575 | [M-H]- | Lactosyl Ceramide (d18:1/16:0), Level 2A | C_46_H_87_NO_13_ |  |
| 17 | 699.5649 | 698.5565 | 5.94 | 0.44  (0.35—0.53) | 1.22E-06 | 0.000175761 | [M-H]- | HEX-Ceramide(d18:1/16:0), LEVEL 2A | C_40_H_77_NO_8_ |  |
| 18 |  | 796.5346 | 5.96 | 0.4  (0.29—0.51) | 1.15E-05 | 0.000239473 |  | UNKNOWN |  |  |
| 19 |  | 757.4997 | 6 | 0.42  (0.32—0.51) | 4.34E-06 | 0.000185234 |  | UNKNOWN |  |  |
| 20 |  | 1277.8086 | 6.22 | 0.37  (0.31—0.42) | 6.35E-05 | 0.000606646 |  | UNKNOWN |  |  |
| 21 |  | 388.0639 | 6.23 | 0.41  (0.34—0.48) | 8.12E-06 | 0.000219088 |  | UNKNOWN |  |  |
| 22 |  | 1251.7924 | 6.23 | 0.37  (0.32—0.41) | 7.02E-05 | 0.000650231 |  | UNKNOWN |  |  |
| 23 |  | 1176.4299 | 6.36 | -0.41  (-0.51—-0.29) | 1.03E-05 | 0.000233031 |  | UNKNOWN |  |  |
| 24 |  | 388.0638 | 6.4 | 0.36  (0.26—0.46) | 9.50E-05 | 0.000792676 |  | UNKNOWN |  |  |
| 25 |  | 962.535 | 6.4 | 0.41  (0.31—0.49) | 1.13E-05 | 0.000239042 |  | UNKNOWN |  |  |
| 26 |  | 572.4809 | 6.47 | 0.39  (0.33—0.46) | 2.03E-05 | 0.000310714 | [M+Cl]- | Ceramide (d18:1/16:0), LEVEL2A | C_34_H_67_NO_3_ | Based on MSMS data |
| 27 |  | 599.5007 | 6.47 | 0.39  (0.3—0.45) | 3.03E-05 | 0.000388487 |  | UNKNOWN |  |  |
| 28 |  | 536.5042 | 6.47 | 0.41  (0.35—0.47) | 7.11E-06 | 0.000212328 | [M-H]- | Ceramide (d18:1/16:0), LEVEL2A | C_34_H_67_NO_3_ | Based on MSMS data |
| 29 |  | 582.5098 | 6.47 | 0.41  (0.35—0.46) | 9.35E-06 | 0.000227843 | [M+FA-H]- | Ceramide (d18:1/16:0), LEVEL2A | C_34_H_67_NO_3_ | Based on MSMS data |
| 30 |  | 596.5255 | 6.47 | 0.41  (0.35—0.46) | 9.76E-06 | 0.000231579 | [M+Hac-H]- | Ceramide (d18:1/16:0), LEVEL2A | C_34_H_67_NO_3_ | Based on MSMS data |
| 31 |  | 804.7987 | 6.51 | 0.36  (0.26—0.44) | 0.000107868 | 0.000875895 |  | UNKNOWN |  |  |
| 32 |  | 744.5541 | 6.51 | 0.36  (0.24—0.45) | 0.000116267 | 0.000928322 |  | UNKNOWN |  |  |
| 33 |  | 1504.1313 | 6.51 | 0.36  (0.24—0.45) | 0.000119258 | 0.000940677 |  | UNKNOWN |  |  |
| 34 | 819.5976 | 818.5903 | 6.52 | 0.36  (0.24—0.45) | 0.000117101 | 0.000932754 | M-H, M+K-2H | UNKNOWN |  |  |
| 35 |  | 1279.8237 | 6.87 | 0.4  (0.34—0.45) | 1.78E-05 | 0.000289726 |  | UNKNOWN |  |  |
| 36 |  | 730.5741 | 6.88 | 0.41  (0.35—0.47) | 7.74E-06 | 0.000212328 |  | UNKNOWN |  |  |
| 37 |  | 842.5655 | 6.88 | 0.4  (0.35—0.46) | 1.15E-05 | 0.000239473 |  | UNKNOWN |  |  |
| 38 |  | 940.548 | 6.88 | 0.41  (0.34—0.47) | 9.83E-06 | 0.000231579 |  | UNKNOWN |  |  |
| 39 |  | 790.5947 | 6.88 | 0.39  (0.32—0.45) | 2.40E-05 | 0.0003417 |  | UNKNOWN |  |  |
| 40 |  | 864.613 | 6.88 | 0.37  (0.29—0.44) | 6.82E-05 | 0.000633986 |  | UNKNOWN |  |  |
| 41 |  | 1060.5034 | 6.88 | 0.38  (0.31—0.44) | 4.81E-05 | 0.000512249 |  | UNKNOWN |  |  |
| 42 |  | 1322.4062 | 6.91 | 0.38  (0.3—0.46) | 3.88E-05 | 0.000450481 |  | UNKNOWN |  |  |
| 43 |  | 1202.4525 | 6.92 | 0.36  (0.27—0.44) | 0.000131418 | 0.0009995 |  | UNKNOWN |  |  |
| 44 |  | 806.6007 | 7.01 | 0.38  (0.28—0.47) | 4.05E-05 | 0.000457591 |  | UNKNOWN |  |  |
| 45 |  | 780.5876 | 7.07 | 0.4  (0.31—0.48) | 1.71E-05 | 0.000284098 |  | UNKNOWN |  |  |
| 46 |  | 627.5321 | 7.15 | 0.4  (0.32—0.46) | 1.68E-05 | 0.000280671 |  | UNKNOWN |  |  |
| 47 |  | 600.5124 | 7.15 | 0.37  (0.32—0.41) | 6.64E-05 | 0.000624575 |  | UNKNOWN |  |  |
| 48 |  | 624.5567 | 7.16 | 0.39  (0.34—0.43) | 3.17E-05 | 0.000398297 |  | UNKNOWN |  |  |
| 49 |  | 610.5412 | 7.16 | 0.38  (0.33—0.42) | 4.59E-05 | 0.000498047 |  | UNKNOWN |  |  |
| 50 |  | 564.5354 | 7.16 | 0.37  (0.33—0.41) | 6.04E-05 | 0.000586011 |  | UNKNOWN |  |  |
| 51 |  | 798.6051 | 7.22 | 0.39  (0.28—0.47) | 3.05E-05 | 0.000389115 |  | UNKNOWN |  |  |
| 52 |  | 858.6246 | 7.22 | 0.36  (0.26—0.46) | 0.000114479 | 0.000918431 |  | UNKNOWN |  |  |
| 53 |  | 806.6301 | 7.42 | 0.44  (0.36—0.51) | 1.75E-06 | 0.000175761 |  | UNKNOWN |  |  |
| 54 |  | 732.5913 | 7.43 | 0.37  (0.28—0.45) | 7.99E-05 | 0.000702052 |  | UNKNOWN |  |  |
| 55 |  | 944.6613 | 7.63 | 0.38  (0.28—0.48) | 4.84E-05 | 0.000513527 |  | UNKNOWN |  |  |
| 56 |  | 653.5358 | 7.67 | 0.38  (0.31—0.45) | 4.70E-05 | 0.000505113 |  | UNKNOWN |  |  |
| 57 |  | 714.6217 | 7.71 | 0.37  (0.27—0.46) | 5.98E-05 | 0.000583649 |  | UNKNOWN |  |  |
| 58 | 240.1714 | 221.1536 | 7.72 | 0.42  (0.33—0.5) | 5.87E-06 | 0.000206829 | M-H_2_O-H, 2M+FA-H | UNKNOWN |  |  |
| 59 |  | 740.6329 | 7.72 | 0.38  (0.28—0.46) | 4.96E-05 | 0.000520016 |  | UNKNOWN |  |  |
| 60 |  | 639.3944 | 7.72 | 0.38  (0.29—0.47) | 5.11E-05 | 0.000527612 |  | UNKNOWN |  |  |
| 61 |  | 235.1206 | 7.72 | 0.36  (0.27—0.45) | 9.49E-05 | 0.000792676 |  | UNKNOWN |  |  |
| 62 |  | 483.2646 | 7.72 | 0.36  (0.28—0.43) | 0.000127287 | 0.000972224 |  | UNKNOWN |  |  |
| 63 |  | 277.1796 | 7.73 | 0.42  (0.33—0.5) | 6.38E-06 | 0.000207112 |  | UNKNOWN |  |  |
| 64 |  | 679.5513 | 7.78 | 0.47  (0.41—0.52) | 2.26E-07 | 0.000126229 |  | UNKNOWN |  |  |
| 65 | 593.5747 | 652.588 | 7.79 | 0.37  (0.34—0.41) | 5.47E-05 | 0.000551445 | [M+CH3COO]- | Ceramide (d18:1/20:0), LEVEL 2A | C_38_H_75_NO_3_ |  |
| 66 | 593.5747 | 592.567 | 7.79 | 0.37  (0.33—0.4) | 8.62E-05 | 0.000739591 | [M-H]- | Ceramide (d18:1/20:0), LEVEL 2A | C_38_H_75_NO_3_ |  |
| 67 |  | 769.6219 | 7.81 | 0.4  (0.31—0.49) | 1.34E-05 | 0.000251247 |  | UNKNOWN |  |  |
| 68 |  | 999.6944 | 7.92 | 0.38  (0.32—0.46) | 3.41E-05 | 0.000413539 |  | UNKNOWN |  |  |
| 69 |  | 974.6668 | 8.25 | 0.38  (0.3—0.45) | 3.65E-05 | 0.000437687 |  | UNKNOWN |  |  |
| 70 |  | 683.5962 | 8.37 | 0.36  (0.25—0.46) | 0.00010742 | 0.00087438 |  | UNKNOWN |  |  |
| 71 |  | 744.5914 | 8.38 | 0.4  (0.34—0.45) | 1.28E-05 | 0.000247349 |  | UNKNOWN |  |  |
| 72 | 621.606 | 656.5749 | 8.38 | 0.37  (0.28—0.45) | 5.55E-05 | 0.00055487 | [M+Cl]- | Ceramide (d18:1/22:0), LEVEL 2A | C_40_H_79_NO_3_ |  |
| 73 | 621.606 | 718.5756 | 8.39 | 0.43  (0.36—0.49) | 3.20E-06 | 0.000185234 | [M+PO_4_H_2_]- | Ceramide (d18:1/22:0), LEVEL 2A | C_40_H_79_NO_3_ |  |
| 74 |  | 771.6378 | 8.43 | 0.41  (0.31—0.5) | 9.49E-06 | 0.000228327 |  | UNKNOWN |  |  |
| 75 |  | 762.6025 | 8.69 | 0.37  (0.29—0.44) | 8.00E-05 | 0.000702052 |  | UNKNOWN |  |  |
| 76 |  | 1011.7436 | 8.73 | 0.41  (0.32—0.5) | 7.42E-06 | 0.000212328 |  | UNKNOWN |  |  |
| 77 |  | 331.1908 | 8.8 | 0.36  (0.31—0.41) | 0.00010074 | 0.000828065 |  | UNKNOWN |  |  |
| 78 |  | 982.7803 | 8.89 | 0.36  (0.29—0.43) | 0.000114886 | 0.000919488 |  | UNKNOWN |  |  |
| 79 |  | 313.1668 | 8.99 | 0.38  (0.29—0.48) | 4.56E-05 | 0.000497021 |  | UNKNOWN |  |  |

Table 6: Confusion matrix based on the cross-validated model displaying the true and false positive and negatives predicted by the model based on data acquired by ^1^H NMR spectroscopy.

|  |  | **Predicted condition** | |
| --- | --- | --- | --- |
|  |  | **0** | **1** |
| **True condition** | **0** | 40 | 8 |
|  | **1** | 14 | 31 |

Table 7: Confusion matrix based on the cross-validated model displaying the true and false positive and negatives predicted by the model based on data acquired by HILIC.

|  |  | **Predicted condition** | |
| --- | --- | --- | --- |
|  |  | **0** | **1** |
| **True condition** | **0** | 50 | 6 |
|  | **1** | 21 | 30 |

Table 8: Confusion matrix based on the cross-validated model displaying the true and false positive and negatives predicted by the model based on data acquired by Lipidomics ESI-.

|  |  | **Predicted condition** | |
| --- | --- | --- | --- |
|  |  | **0** | **1** |
| **True condition** | **0** | 51 | 6 |
|  | **1** | 23 | 32 |

Table 9: Confusion matrix based on the cross-validated model displaying the true and false positive and negatives predicted by the model based on data acquired by Lipidomics ESI+.

|  |  | **Predicted condition** | |
| --- | --- | --- | --- |
|  |  | **0** | **1** |
| **True condition** | **0** | 49 | 8 |
|  | **1** | 18 | 37 |

# Supplementary methods

**Case definitions**

We used the WHO Definitions and reporting framework for tuberculosis. The NIH criteria were not used as they explicitly exclude children with known household contact with TB.

The WHO definitions and their application in these study cohorts are summarised here:

‘Presumptive TB refers to a patient who presents with symptoms or signs suggestive of TB (previously known as a TB suspect).

A bacteriologically confirmed TB case is one from whom a biological specimen is positive by smear microscopy, culture or WHO-approved rapid diagnostics (such as Xpert MTB/RIF).

A clinically diagnosed TB case is one who had suggestive symptoms and signs of TB, did not fulfil the criteria for bacteriological confirmation but had suggestive appearance on chest radiograph, failed to respond to empirical broad-spectrum antibiotics and had favourable response to specific anti-tuberculous therapy +/− positive tuberculin skin test +/− suggestive histological appearances on biopsy material. This definition includes cases diagnosed on the basis of X-ray abnormalities or suggestive histology and extrapulmonary cases without laboratory confirmation. Clinically diagnosed cases subsequently found to be bacteriologically positive (before or after starting treatment) were reclassified as bacteriologically confirmed.’ ^4,5^ Chest radiographs were interpreted by the responsible clinical teams in Gambia (at least two clinicians) or clinical radiologists in the UK who were not blinded to the clinical context but were not involved in any laboratory analyses..

The criteria used for symptoms suggestive of TB were: cough>2 weeks, weight loss or failure to gain weight, fever, malaise/fatigue, neck swelling, haemoptysis, night sweats, and wheeze.

# Mass spectrometry analysis

**Sample sorting, formatting, and aliquoting.**

Serum samples were stored at -80°C and remained frozen while being sorted according to the established analysis order, avoiding confounding critical clinical variables with analytical run order effects. Once sorted, samples were thawed overnight at 4°C, vortexed, centrifuged at 3486 × g for 10 minutes at 4°C (Eppendorf 5810R centrifuge) and aliquoted into 0.5 mL 96-well plates including 50 µL for lipidomic profiling, and 100 µL for HILIC. All plates were sealed with a silicone cap mat and returned to -80°C storage. Columns 11 and 12 of the plates are left empty for adding the pooled quality control samples.

Where sample volume was too low to allow analysis by all methods, we prioritised analysis by mass spectrometry over ^1^H NMR spectroscopy. In terms of mass spectrometry, the lipidomic analysis was prioritised over HILIC.

**Study reference (SR), and SR dilution**

Data quality within each experiment was assessed using SR samples. A pooled SR sample was prepared during the aliquoting step. The HILIC sub-fractions were supplemented with the HILIC method reference (MR) mixture in Table 10 in a 2:1 SR:MR ratio, homogenized, and aliquoted into 1.7 mL polypropylene microcentrifuge tubes. All SR materials were frozen at -80°C until required for analytical plate preparation.

Table 10: HILIC MR standards

| No. | Standard | Supplier & P/N |
| --- | --- | --- |
| 1 | Phenylalanine-^13^C_9_,^15^N | Sigma, 608017 |
| 2 | Adenine-2d_1_ | CDN isotopes, D-6291 |
| 3 | Taurine-^15^N | Sigma, 605956 |
| 4 | Creatine-d_3_.H_2_O | Sigma, 616249 |
| 5 | Arginine-^13^C_6_ | Sigma, 643440 |
| 6 | Tryptophan-d_5_ | Sigma, 615862 |
| 7 | Uracil-2-^13^C,^15^N_2_ | Sigma, 608459 |

A dilution series of SR samples (1%, 10%, 20%, 40%, 60%, 80% and 100% original concentration) was prepared for each assay type from thawed SR in the same manner as the study samples and stored in polypropylene vials for analysis supporting multiple injections from the same stock. Dilution series samples were analysed prior to and following the analysis of the samples.

**Sample preparation for UPLC-MS analysis**

Serum samples (100 µL) were thawed at 4°C for 2 hours and subsequently prepared for HILIC profiling by addition of 10 µL of water containing HILIC internal standard as indicated in Table 11, and 300 µL of acetonitrile for protein precipitation. Plates were heat sealed (Thermo-Seal heat sealing foil sheets) using a ALPS 50 V-Manual Heat Sealer (Thermo SCIENTIFIC) prior to mixing using a MixMate (Eppendorf) operating at 1400 rpm for 2 hours at 4°C. The plates were subsequently centrifuged at 4°C for 10 minutes at 3486 × g to separate precipitated protein from the homogenous supernatant. The supernatant (125 µL) was aspirated using an eight-channel 15-1200 µL Eppendorf Xplorer Plus pipette and dispensed into a 350 µL 96-well plate, for positive mode ESI profiling. Plates were heat sealed for analysis, and centrifugation was repeated for 5 minutes at 4°C and 3486 × g. An additional 25 µL of supernatant was taken from each sample and pooled to create a SR sample which was prepared with MR and injected repeatedly every 11 samples throughout the analysis to assess data quality.

Serum samples (50 µL) were thawed at 4°C for 2 hours and subsequently prepared for lipidomic profiling by addition of 400 µL of 2-propanol containing a mixture of reference standards as indicated in Table 11. Plates were heat sealed (Thermo-Seal heat sealing foil sheets) using a ALPS 50 V-Manual Heat Sealer prior to mixing using a MixMate operating at 1400 rpm for 2 hours at 4°C. The plates were subsequently centrifuged at 4°C for 10 minutes at 3486 × g to separate precipitated protein from the homogenous supernatant. The supernatant was aspirated using an eight-channel 15-1200 µL Eppendorf Xplorer Plus pipette and dispensed into two separate 350 µL 96-well plates (100 µl in each), for positive and negative mode ESI profiling. Plates were heat sealed for analysis, and centrifugation was repeated for 5 minutes at 4°C and 3486 × g. The additional supernatant (50 µL) was removed from each sample and pooled to create a SR sample which injected repeatedly every 11 samples throughout the analysis to assess data quality.

Table 11: Standard mixtures for HILIC and lipidomics

| No. | Standard | Supplier & P/N |
| --- | --- | --- |
| HILIC mixture | | |
| 1 | N-Benzoyl-d5-glycine | CDN isotopes, D-5588 |
| 2 | Adenosine-2-d-1 | CDN isotopes, D-1827 |
| Lipid mixture | | |
| 1 | LPC9 | Avanti, 855276P |
| 2 | PC11 | Avanti, 850330P |
| 3 | C17 | Sigma, H3500 |
| 4 | PG15 | Avanti, 840446P |
| 5 | PE15 | Avanti, 850704P |
| 6 | PS17 | Avanti, 840028P |
| 7 | PA17 | Avanti, 830856P |
| 8 | Cer17 | Avanti, 860517P |
| 9 | DG19 | Sigma, 68633 |
| 10 | PC23 | Avanti, 850372P |
| 11 | TG15 | Sigma, T4257 |
| 12 | TG17 | Sigma, T2151 |

**Chromatographic conditions**

HILIC chromatography was conducted using an Acquity BEH HILIC (1.7 µm, 2.1 × 150 mm) column (Waters Corporation, Milford, U.S.A.) thermostated at 40 °C by a Waters Acuity UPLC system (Waters Corporation, Milford, MA, USA). Gradient elution was used for separation, using 0.1% (v/v) formic acid and 20 mM ammonium formate in high-grade LC-MS water (Fisher) for mobile phase A and 0.1% (v/v) formic acid in high-grade LC-MS acetonitrile (Sigma) for mobile phase B at a flow rate of 0.6 ml/min. A 15-minute gradient was performed: starting conditions 95% B were kept for 0.1 min followed by a first decrease to 80% B from 0.1 to 4.60 min and a second decrease to 50% B from 4.6 to 5.50 min. These conditions were kept until 7.00 min before the gradient was set back to initial conditions of 95% B at 7.1 min that remained until the end of the runtime. Full loop 2 µL injections (5× overfill) of prepared samples were made into the chromatographic system.

Lipid profiling employed an Acquity BEH C8 (1.7 µm, 2.1 × 100 mm) column (Waters Corporation, Milford, U.S.A.) thermostated at 40 °C by a Waters Acquity UPLC system (Waters Corporation, Milford, MA, USA). Gradient elution with a flow rate of 0.6 mL/min was used for separation. Mobile phase A consisted of the following mixture: water/isopropanol/acetonitrile (all high-grade LC-MS from Fisher or Sigma) in a 50:25:25 ratio with the addition of 5 mM ammonium acetate, 0.05% acetic acid, and 20 µM phosphoric acid (Sigma). Mobile phase B consisted of isopropanol/acetonitrile (both high-grade LC-MS solvents from Sigma) in a ratio of 50:50 with 5 mM ammonium acetate and 0.05% acetic acid as modifiers. A 15-minute gradient was performed: starting conditions of 1% B were kept for 0.1 min followed by an increase to 30% from 0.1 to 2.0 min and to 90% B till 11.5 min. At 12 min 99.9% B was used for washing out until 12.55 min. After that, initial conditions were re-established for the remaining runtime. Full loop 2 µl injections (5× overfill) of prepared samples were made into the chromatographic system.

**Mass spectrometry**

Mass spectrometry was performed on high-resolution Q-Tof instruments (Xevo G2-XS) from Waters (Ltd., Manchester, UK) in both positive and negative ion electrospray ionization (ESI + and ESI–) modes. The MS parameters were set as follows: capillary voltage 1.0-2.0 kV, sample cone voltage 20-25 V, source offset 80, source temperature 120°C, desolvation temperature 600°C, desolvation gas flow 1000 L/h, and cone gas flow 150 L/h. Data were collected in centroid mode with a scan range of 50-1200 m/z for small molecule profiling (HILIC chromatography) and of 50–2000 m/z for lipidomic profiling and a scan time of 0.07 and 0.15 s, respectively. For mass accuracy, LockSpray mass correction was employed using a 200 pg/μL leucine enkephalin (m/z 556.2771 in ESI+, m/z 554.2615 in ESI−) solution in water/acetonitrile (50:50) at a flow rate of 10 μL/min. Lockmass scans were collected every 60 s and averaged over 3 scans as advised by the manufacturer.

**^1^H NMR spectroscopic analysis**

A 600MHz spectrometer (Bruker, Germany) with a probe temperature of 310K was used. Samples underwent a standard 1D pulse sequence to suppress the water signal using an acquisition pulse sequence using the first increment of the nuclear Overhauser effect (NOE) parameters, defined in ^6^. Spectra were acquired using 32 scans and four dummy scans. J-resolved (JRES) spectra were also collected for each sample. A QC pool was made by pooling 50μl of each sample; these were analysed in the same manner as the study samples. A QC sample also underwent 2D experiments to assist in metabolite assignment, including total correlation spectroscopy (TOCSY), correlation spectroscopy (COSY) and heteronuclear single-quantum correlation spectroscopy (HSQC).

# Metabolite Identification

For ^1^H NMR spectroscopic data, metabolites were identified using their chemical shifts (δ) and peak splitting patterns, which were compared against the Human Metabolome Database (HMDB, http://www.hmdb.ca/). Also, Statistical Total Correlation Spectroscopy (STOCSY) was used to aid in metabolite identification ^7^. Further 2D experiments were used to compare the carbon and hydrogen resonances to the HMDB database.

For metabolite identification, mass to charge (*m/z*) values were searched against online databases, METLIN (https://metlin.scripps.edu), LIPID MAPS (www.lipidmaps.org), and HMDB. Metabolites were identified by inspecting the fragmentation patterns in the MS/MS data acquired for the presence of head groups, increasing the confidence in the assignment.

# References

1 Eriksson L, J. E., Kettaneh-Wold N, Wold S. *Introduction to multi- and megavariate data analysis using projection methods (PCA & PLS)* 213–225 (Umetrics, 1999).

2 Umetrics. *SIMCA –P and Multivariate Analysis*, <<https://umetrics.com/sites/default/files/kb/multivariate_faq.pdf>> (

3 Dieterle, F., Ross, A., Schlotterbeck, G. & Senn, H. Probabilistic quotient normalization as robust method to account for dilution of complex biological mixtures. Application in H-1 NMR metabonomics. *Anal Chem* **78**, 4281-4290, doi:10.1021/ac051632c (2006).

4 Definitions and reporting framework for tuberculosis: 2013 revision. (World Health Organization, Geneva, Switzerland, 2013).

5 Togun, T. O. *et al.* No added value of interferon-gamma release to a prediction model for childhood tuberculosis. *Eur Respir J* **47**, 223-232, doi:10.1183/13993003.00890-2015 (2016).

6 Dona, A. C. *et al.* Precision high-throughput proton NMR spectroscopy of human urine, serum, and plasma for large-scale metabolic phenotyping. *Anal Chem* **86**, 9887-9894, doi:10.1021/ac5025039 (2014).

7 Cloarec, O. *et al.* Statistical total correlation spectroscopy: an exploratory approach for latent biomarker identification from metabolic 1H NMR data sets. *Anal Chem* **77**, 1282-1289, doi:10.1021/ac048630x (2005).
